# Circulating mitochondria promoted endothelial cGAS-derived neuroinflammation in subfornical organ to aggravate sympathetic overdrive in heart failure mice

**DOI:** 10.1186/s12974-024-03013-x

**Published:** 2024-01-19

**Authors:** Shutian Zhang, Dajun Zhao, Zhaohua Yang, Fanshun Wang, Shouguo Yang, Chunsheng Wang

**Affiliations:** 1 Department of Cardiac Surgery, Zhongshan Hospital, Fudan University, Shanghai, 200032 China; 2 Shanghai Institute of Cardiovascular Diseases, Shanghai, 200032 China

**Keywords:** Circulating mitochondria, Heart failure, Sympathoexcitation, cGAS, DHODH, Subfornical organ, Neuroinflammation, Endothelial cells

## Abstract

**Background:**

Sympathoexcitation contributes to myocardial remodeling in heart failure (HF). Increased circulating pro-inflammatory mediators directly act on the Subfornical organ (SFO), the cardiovascular autonomic center, to increase sympathetic outflow. Circulating mitochondria (C-Mito) are the novel discovered mediators for inter-organ communication. Cyclic GMP–AMP synthase (cGAS) is the pro-inflammatory sensor of damaged mitochondria.

**Objectives:**

This study aimed to assess the sympathoexcitation effect of C-Mito in HF mice via promoting endothelial cGAS-derived neuroinflammation in the SFO.

**Methods:**

C-Mito were isolated from HF mice established by isoprenaline (0.0125 mg/kg) infusion via osmotic mini-pumps for 2 weeks. Structural and functional analyses of C-Mito were conducted. Pre-stained C-Mito were intravenously injected every day for 2 weeks. Specific cGAS knockdown (cGAS KD) in the SFO endothelial cells (ECs) was achieved via the administration of AAV9-TIE-shRNA (cGAS) into the SFO. The activation of cGAS in the SFO ECs was assessed. The expression of the mitochondrial redox regulator Dihydroorotate dehydrogenase (DHODH) and its interaction with cGAS were also explored. Neuroinflammation and neuronal activation in the SFO were evaluated. Sympathetic activity, myocardial remodeling, and cardiac systolic dysfunction were measured.

**Results:**

C-Mito were successfully isolated, which showed typical structural characteristics of mitochondria with double-membrane and inner crista. Further analysis showed impaired respiratory complexes activities of C-Mito from HF mice (C-Mito^HF^) accompanied by oxidative damage. C-Mito entered ECs, instead of glial cells and neurons in the SFO of HF mice. C-Mito^HF^ increased the level of ROS and cytosolic free double-strand DNA (dsDNA), and activated cGAS in cultured brain endothelial cells. Furthermore, C-Mito^HF^ highly expressed DHODH, which interacted with cGAS to facilitate endothelial cGAS activation. C-Mito^HF^ aggravated endothelial inflammation, microglial/astroglial activation, and neuronal sensitization in the SFO of HF mice, which could be ameliorated by cGAS KD in the ECs of the SFO. Further analysis showed C-Mito^HF^ failed to exacerbate sympathoexcitation and myocardial sympathetic hyperinnervation in cGAS KD HF mice. C-Mito^HF^ promoted myocardial fibrosis and hypertrophy, and cardiac systolic dysfunction in HF mice, which could be ameliorated by cGAS KD.

**Conclusion:**

Collectively, we demonstrated that damaged C-Mito^HF^ highly expressed DHODH, which promoted endothelial cGAS activation in the SFO, hence aggravating the sympathoexcitation and myocardial injury in HF mice, suggesting that C-Mito might be the novel therapeutic target for sympathoexcitation in HF.

**Graphic Abstract:**

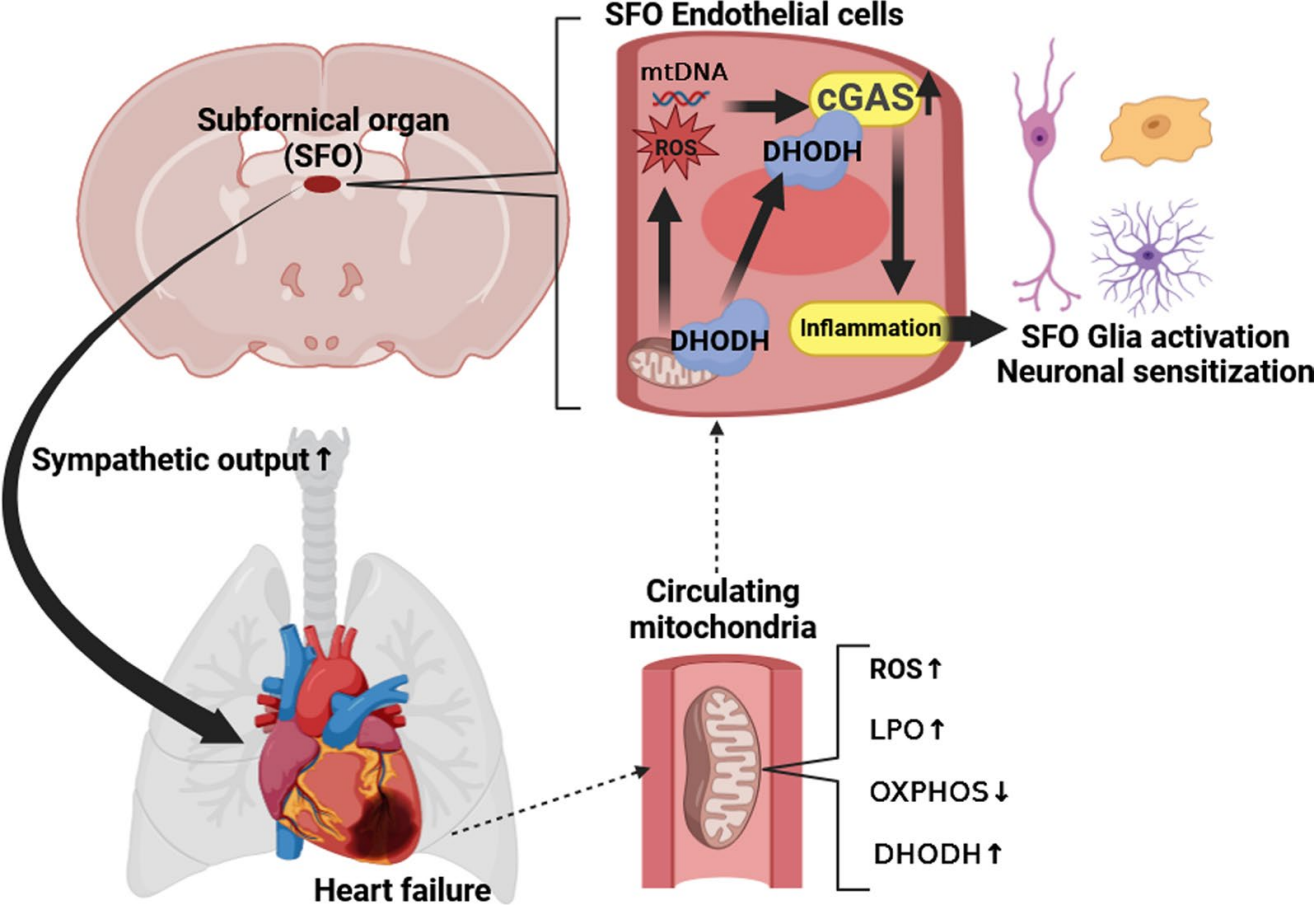

**Supplementary Information:**

The online version contains supplementary material available at 10.1186/s12974-024-03013-x.

## Highlights

- Circulating mitochondria from HF mice (C-Mito^HF^) were respiratory impaired and oxidatively damaged.
- C-Mito^HF^ entered the endothelial cells of the SFO in HF mice, promoted ROS accumulation and cytosolic free dsDNA release, and further activated endothelial cGAS in the SFO.
- Dihydroorotate dehydrogenase (DHODH), a mitochondrial redox regulator, was highly expressed in C-Mito^HF^, which directly interacted with cGAS in the endothelial cells to promote cGAS activation.
- C-Mito^HF^-inducing endothelial cGAS activation in the SFO aggravated sympathoexcitation and myocardial remodeling in HF mice.

**Supplementary Information:**

The online version contains supplementary material available at 10.1186/s12974-024-03013-x.

## Introduction

Heart failure (HF) is a global public health challenge [1]. A variety of cardiovascular diseases, including hypertension, coronary heart disease, and valvular disease, eventually lead to HF [2–7]. Sympathetic hyperactivation, a key contributor to HF development, induces myocardial remodeling by overactivating cardiac adrenergic receptors, resulting in pathological cardiomyocyte hypertrophy and myocardial fibrosis [8–15]. The neural cells in the sympathetic regulating center, including the subfornical organ (SFO), paraventricular nucleus (PVN), rostral ventrolateral medulla (RVLM), and thoracolumbar intermediolateral column of the spinal cord, directly influence the sympathetic tone of the heart and blood vessels [16–22]. However, researchers have not found a therapeutic target for the dysregulation of the sympathetic center due to the existence of the blood–brain barrier.

Notably, the SFO lacks a complete blood–brain barrier, enabling its endothelial cells to sense circulating inflammatory stimuli and convert them into local brain signals [23–28]. Extracellular substances originating from the heart and vessels can influence the sympathetic center through circulation, thereby promoting sustained enhancement of cardiac sympathetic activity and exacerbating the progression of HF [29–31]. These blood-derived elements, including exosomes and extracellular vesicles, carry microRNAs, proteins, reactive oxygen species (ROS), and other chemicals that intensify neuroinflammation and oxidative stress in the sympathetic center of HF animal models [32]. Targeting these blood-derived substances emerges as a promising strategy to alleviate sympathetic center dysregulation and mitigate pathological sympathoexcitation in HF [33–42].

Recent studies have shown that mitochondria could be actively released into the extracellular space, and then taken up by neighboring cells, affecting the energy metabolism and signal transduction of recipient cells [43–47]. These functional extracellular mitochondria can enter circulation, now recognized as circulating mitochondria (C-Mito) [48–50]. C-Mito are newly confirmed as a novel mediator for inter-organ communication. Adipocyte-derived C-Mito could enter cardiomyocytes and significantly induce preconditioning of oxidative stress insult, thus protecting cardiomyocytes from myocardial ischemia/reperfusion (MI/R) injury [51]. Upon entering into the cytoplasm of recipient cells, C-Mito can activate signaling pathways such as cyclic GMP–AMP synthase (cGAS), stimulator of interferon genes protein (STING), NLR family pyrin domain-containing 3 (NLRP3), potentially inducing inflammation [52–55]. Given the crucial role of mitochondria in the normal physiological functions of the heart and brain, it remains unexplored whether C-Mito contribute to the heart–brain communication under the HF progression.

cGAS is a molecular sensor for damaged mitochondria [56, 57]. The release of free mitochondrial DNA (mtDNA) serves as a key activator of cGAS. cGAS not only regulates the production of type I interferon and inflammatory cytokines through the classical STING pathway but also strongly promotes ROS generation, leading to oxidative cell damage [58, 59]. In the context of myocardial injury, cGAS activation has been linked to excessive endoplasmic reticulum (ER) stress, apoptosis, and inflammatory infiltration in animal models of ischemic cardiomyopathy, reperfusion myocardial injury, and hypertrophic cardiomyopathy [60–64]. Recent studies reveal that extracellular mitochondria can enter myocardial microvascular endothelial cells, up-regulating endothelial intercellular adhesion molecule 1 (ICAM) expression through the cGAS/STING signaling pathway. This, in turn, recruits inflammatory cells, exacerbating organ rejection [65, 66]. Extracellular mitochondria can also activate cGAS in cardiac fibroblasts, contributing to myocardial fibrosis in MI/R injury [67]. However, it remains unexplored whether C-Mito can activate central cGAS and participate in the sympathoexcitation in HF. In the present study, we aim to explore the pathological role of C-Mito in central sympathetic overactivation in HF and its underlying mechanisms.

## Materials and methods

### Drugs and reagents

Isoprenaline (ab120710), Teriflunomide (ab141480), and RU.521 (ab287088) were obtained from Abcam (Cambridge, UK). AAV9-TIE-shRNA (Scramble) and AAV9-TIE-shRNA (cGAS) were purchased from Hanbio Co. Ltd. (Shanghai China). Mitochondria Storage Buffer (C3609) was purchased from Beyotime (Shanghai, China). Pierce Protein A/G Agarose (20421) was provided by Thermo Fisher Scientific (Waltham, USA). Wheat germ agglutinin (W11261) was purchased from Thermo Fisher Scientific (Waltham, USA). DAPI (C1002) was obtained from Beyotime (Shanghai, China). The following antibodies CD31 (ab222783, 1:500 for immunofluorescent staining), β-actin (ab179467, 1:5000 for immunoblotting), TNF-α (ab215188, 1:200 for immunofluorescent staining), IL-1β (ab254360, 1:200 for immunofluorescent staining), Iba-1 (ab283319, 1:100 for immunofluorescent staining), iNOS (ab178945, 1:200 for immunofluorescent staining), GFAP (ab279289, 1:200 for immunofluorescent staining), NeuN (ab279296, 1:200 for immunofluorescent staining), c-fos (ab222699, 1:500 for immunofluorescent staining), TH (ab137869, 1:200 for immunofluorescent staining), GAP43 (ab75810, 1:200 for immunofluorescent staining), cTnT (ab8295, 1:200 for immunofluorescent staining), Total OXPHOS Rodent Antibody Cocktail (ab110413), Hsp60 (ab190828, 1:1000 for immunoblotting), COX IV (ab202554, 1:1000 for immunoblotting), Goat Anti-Chicken IgY H&L (Alexa Fluor 647) (ab150171, 1:500 for immunofluorescent staining), Donkey Anti-Rabbit IgG H&L (Alexa Fluor 647) (ab150075, 1:500 for immunofluorescent staining), Donkey Anti-Rabbit IgG H&L (Alexa Fluor 488) (ab150073, 1:500 for immunofluorescent staining), Donkey Anti-Rabbit IgG H&L (HRP) (ab6802, 1:1000 for immunoblotting), Goat Anti-Mouse IgG H&L (Alexa Fluor 488) (ab150113, 1:500 for immunofluorescent staining), Rabbit Anti-Mouse IgG H&L (HRP) (ab6728, 1:1000 for immunoblotting), Goat Anti-Mouse IgG H&L (Alexa Fluor 647) (ab150115, 1:500 for immunofluorescent staining) were purchased from Abcam (Cambridge, UK). The following antibodies cGAS (PA5-141097, 1:1000 for immunoblotting, 1:30 for immunoprecipitation), dsDNA (MA1-35346, 1:500 for immunofluorescent staining), TOM20 (MA5-24859, 1:500 for immunofluorescent staining, 1:1000 for immunoblotting), C3a (PA1-30601, 1:500 for immunofluorescent staining), and TIM50 (MA5-37851, 1:1000 for immunoblotting) were purchased from Thermo Fisher Scientific (Waltham, USA). The following kits 2′3′-Cyclic GAMP ELISA Kit (EIAGAMP, thermo), MitoSox Green & MitoSox Red (M26009), BODIPY 581/591 probe (D3861 THERMO), and IL-1β Mouse ELISA Kit (Thermo BMS6002) were purchased from Thermo Fisher Scientific (Waltham, USA). The following kits EZQuant dsDNA Quantitation kit (ab287882), MitoTox™ Complete OXPHOS Activity Assay Kit (ab110419), Mouse TNF-α ELISA Kit (ab208348), Masson Stain Kit (ab150669) were purchased from Abcam (Cambridge, UK). The following kits DCFH-DA (S0033M), Mitochondrial membrane potential assay kit with JC-1 (C2006), MitoTracker Green (C1048), and MitoTracker Red (C1049) were purchased from Beyotime (Shanghai, China). Mouse NT-proBNP ELISA Kit (E-EL-M0834c) was obtained from Elabscience (Wuhan, China). The Mouse NE ELISA Assay kit (NOU39-K010) was provided by Eagle Biosciences (Amherst, NH).

### Establishing HF mice and experimental design

We used 6- to 8-week-old male C57BL/6 mice weighing 18 to 22 g in this study. Female mice were excluded from the study because periodic changes in estrogen can affect myocardial function, ultimately leading to increased heterogeneity in experimental results. All mice were purchased from the Laboratory Animal Center of Fudan University. All animals were fed ad libitum with food and water, and housed in a temperature-control room with 24 °C and a 12-h light/12-h dark circadian cycle. Every effort was made to minimize the number and suffering of animals used in this study.

Experiment 1: To explore the functional differences between C-Mito^Ctrl^ and C-Mito^HF^ in mice. Mice were randomly divided into two groups: (1) Ctrl group; (2) HF group; The mice were preacclimated to the feeding environment for 1 week before ISO infusion. A mouse model of HF was established by subcutaneous infusion of ISO (0.0125 mg/kg) via implanted Alzet osmotic mini-pumps for 2 weeks based on the previous reports [68, 69], while the control group of mice was infused with the same amount of saline via implanted Alzet osmotic mini-pumps every day. After 2 weeks of ISO infusion, C-Mito were isolated for identification and functional evaluation. Cardiac function, myocardial remodeling, and sympathetic activity of different groups of mice were detected.

Experiment 2: To investigate the distribution of C-Mito in the sympathetic center and its biological effect on the ECs of the SFO in mice. Mice were randomly divided into four groups: (1) Ctrl mice + C-Mito^Ctrl^ infusion; (2) Ctrl mice + C-Mito^HF^ infusion; (3) HF mice + C-Mito^Ctrl^ infusion; (4) HF mice + C-Mito^HF^ infusion. To determine the biological effect of C-Mito, an equal volume of C-Mito^Ctrl^ and C-Mito^HF^ were infused into the circulating blood of mice. First, C-Mito were pre-labeled with Mito-Tracker before infusion to achieve fluorescent tracing. The same volume (500 μL, 5 × 10^4^) of fluorescently labeled C-Mito^Ctrl^ and C-Mito^HF^ were injected through the tail vein of the mice. The quantity and cellular localization of C-Mito^Ctrl^ and C-Mito^HF^ in the SFO were detected by immunofluorescent staining. The effect of C-Mito^Ctrl^ and C-Mito^HF^ on the expression and activity of cGAS in the SFO were detected by double-labeled immunofluorescent staining and immunoblotting. Double-immunofluorescent staining was used to detect the cellular localization of cGAS in the SFO of mice.

Experiment 3: To investigate the molecular mechanism of C-Mito activating cGAS and the following inflammatory response in cultured brain endothelial cells. The murine brain EC line bEnd.3 was cultured in vitro. The cells were divided into seven groups: (1) control group; (2) C-Mito^Ctrl^-treated group; (3) C-Mito^HF^-treated group; (4) C-Mito^Ctrl^ + RU.521 group; (5) C-Mito^HF^ + RU.521 group; (6) C-Mito^Ctrl^ + Teriflunomide group; (7) C-Mito^HF^ + Teriflunomide group. RU.521 is a specific inhibitor of cGAS. Teriflunomide is a specific inhibitor of DHODH. An equal amount (200 μL, 5 × 10^4^) of C-Mito^Ctrl^ and C-Mito^HF^ pre-labeled with Mito-Tracker were added to the ECs culture medium. In the experiments of group (6) and group (7), C-Mito^Ctrl^ and C-Mito^HF^ were pre-treated with Teriflunomide (5 μM, 15 min) to attenuate DHODH in C-Mito. DCFH-DA and Mito-SOX probes were used to detect the level of reactive oxygen species (ROS) in each group. Double-immunofluorescent staining with anti-dsDNA and anti-TOM20 was used to detect the amount of extra-mitochondrial dsDNA in the cytoplasm. The content of dsDNA in the cytoplasm of each group of cells after mitochondrial removal was directly quantified by the EZQuant dsDNA Quantitation kit. The expression of cGAS was detected by immunoblotting. cGAMP ELISA kit was used to detect the level of cGAMP in each group of cells to evaluate the activation of cGAS. The level of DHODH in C-Mito^Ctrl^ and C-Mito^HF^ was determined by immunoblotting. Double-immunofluorescent staining was used to detect the co-localization of DHODH and cGAS in ECs. Co-immunoprecipitation was used to verify the direct interaction between DHODH and cGAS. TNF-α and IL-1β ELISA kits were used to detect the production of pro-inflammatory cytokines in each group of cells.

Experiment 4: To determine the pathological effects of C-Mito^HF^-inducing cGAS in SFO endothelial cells on neuroinflammation, sympathetic hyperactivation, and myocardial remodeling in HF mice. Mice were divided into the following five groups: (1) Ctrl mice; (2) HF mice; (3) HF mice + C-Mito^Ctrl^; (4) HF mice + C-Mito^HF^; (5) HF mice + C-Mito^HF^ + specific cGAS KD in the ECs of the SFO. Specific cGAS KD in the ECs of the SFO was achieved by injection of AAV9-TIE-shRNA (cGAS) into the SFO of mice. The efficacy of specific cGAS KD in the ECs of the SFO was first verified. Double-immunofluorescent staining was used to detect the expression of pro-inflammatory cytokines in the endothelial cells, inflammatory activation of microglia and astrocytes, and neuronal sensitization in the SFO of mice. Renal sympathetic nerve activity (RSNA) was recorded to directly evaluate the level of cardiovascular sympathetic output in each group of mice. Heart rate variability analysis was used to evaluate the changes in cardiac sympathetic activity in each group. Immunofluorescent staining was used to detect sympathetic innervation and sprouting in the myocardium of mice in each group. MASSON staining and WGA staining were used to evaluate myocardial remodeling in each group of mice. Cardiac function was evaluated by echocardiography. Lung wet weight/dry ratio was used to evaluate the pulmonary congestion associated with left heart failure in each group of mice. The level of plasmic NT-proBNP was measured to evaluate the severity of HF in each group of mice.

### C-Mito isolation and evaluation

#### C-Mito isolation

According to previous studies [48, 50, 51, 66], centrifugation at 1200×*g* for 5 min at 4 °C was conducted to clear cellular debris in plasma samples from Control mice and HF mice. Further centrifugation at 12000×*g* for 5 min at 4 °C was performed to pellet extracellular microparticles containing C-Mito.

Authenticated by immunoblotting results of mitochondrial respiratory complexes proteins (Complex I–V) are shown in Additional file 1: Fig. S1A. Also, the expression of TOM20 (the mitochondrial outer membrane protein), TIM50 (the mitochondrial inner membrane protein), and Hsp60 (the mitochondrial matrix protein) were detected by immunoblotting (Additional file 1: Fig. S1B).

#### Transmission electron microscopy

In brief, for embedding in electron microscopy (EM), C-Mito were poured into flasks and Karnovsky’s fixative (2% paraformaldehyde in 0.1 M phosphate-buffered saline plus 2.5% glutaraldehyde, pH 7.2–7.4). The depth of addition is about 5 mm. C-Mito were then fixed, permeabilized, rinsed with phosphate-buffered saline, dehydrated and embedded in epoxy resin, and polymerized at 70 °C for 24 h. Blocks containing C-Mito were sectioned using an ultramicrotome (Ultracut; Leica) at 70–80 nm. Thin sections were collected on grids and stained with uranyl acetate and lead citrate. Grids were examined under a transmission electron microscope (H-700; Hitachi, Tokyo, Japan) at 80 kV.

#### Nanoparticle tracking analysis

Nanoparticle tracking analysis (NTA) is a widely used and reliable method to measure the particle size of extracellular structures such as extracellular/circulating mitochondria, extracellular vesicles, and exosomes. In previous studies of C-Mito, transmission electron microscopy (TEM) examination combined with NTA particle size analysis was used to confirm the successful isolation of extracellular free mitochondria from circulating blood [48–50]. On the one hand, using TEM, researchers could clearly observed the structure of extracellular free mitochondria, while NTA particle size analysis is a reliable method to detect the particle size of extracellular free mitochondria. Thus, TEM and NTA were used to confirm the isolation of C-Mito from mice in the present study. Ultrapure water was used to rinse Electrophoresis and Brownian Motion Video Analysis Laser Scattering Microscopy. After the instrument stops washing, dilute the standard solution of 100 nm polystyrene microspheres by 250,000 times with ultrapure water, and take 1 mL of the diluted standard solution for automatic use of the instrument. Dilute the C-Mito sample with a clean mitochondrial preserving solution, so that the number of particles displayed in the instrument detection interface of the C-Mito sample is between 50 and 400, preferably around 200. Input the dilution factor of the sample on the software interface, and observe whether the number of particles displayed at the detection position is 50–400. After confirming that the number of particles displayed at each detection position is within the range, click Measurement and Run Video Acquisition on the software interface to start the test. The instrument automatically completes the test process, automatically analyzes the data, and then automatically generates a test report.

#### Mitochondrial membrane potential analysis

Freshly isolated 5 × 10^4^ C-Mito were plated in tubes in a volume of 0.9 mL JC-1 working solution containing MAS 1 × (70 mM sucrose, 220 mM mannitol, 10 mM KH_2_PO_4_, 5 mM MgCl_2_, 2 mM HEPES, 1 mM EGTA, and 0.2% fatty acid-free BSA, pH 7.2) supplemented with 10 mM pyruvate, 2 mM malate and 4 µM JC-1. Tubes were then centrifuged for 20 min at 2000×*g* at 4 °C. After centrifugation, 155 µL of electron flow substrate-containing 1 × MAS was added to each tube, and the tube was warmed up in a 37 °C non-CO_2_ incubator for 10 min. Detect with a fluorescence spectrophotometer or a fluorescence microplate reader: directly perform a time scan with a fluorescence spectrophotometer, the excitation wavelength is 485 nm, and the emission wavelength is 590 nm. When detecting JC-1 monomer, set the wavelength of excitation light to 490 nm and the wavelength of emission light to 530 nm. When detecting JC-1 polymer, set the wavelength of excitation light to 525 nm and the wavelength of emission light to 590 nm.

#### Mitochondrial ROS and lipid peroxides detection

Mitochondrial ROS detection of C-Mito from different groups of mice was assayed using kits containing Mito-SOX probes and BODIPY 581/591 probes according to the manufacturer’s instructions.

#### Respiratory complexes’ activity analysis

The respiratory complexes’ activity of C-Mito from different groups of mice was assayed using MitoTox™ Complete OXPHOS Activity Assay Kit according to the manufacturer’s instructions.

### Specific endothelial cGAS KD in the SFO of mice

Specific endothelial cGAS KD was achieved by injecting AAV9-TIE-shRNA (cGAS) into the SFO of mice. Under isoflurane anesthesia (approximately 2%), AAV9-TIE-shRNA (cGAS) was injected into the SFO of mice using a mouse stereotaxic apparatus. Animals were intubated through a non-invasive polyethylene tube and spontaneously breathed ambient air. The head of the mouse was mounted in a stereotaxic apparatus (Neurostar Tübingen, Germany) and bent at an angle of approximately 45°. After the skull was carefully removed, a glass micropipette was inserted into the SFO (from bregma: AP − 0.48 mm, DV 2.4 mm, ML 0 mm) according to the Paxinos and Franklin atlas. After microinjection, suture the muscle layer covering the fourth ventricle. Maintain the body temperature of the mouse at 37 °C with a heating pad until the animal recovers from surgery. Mice were sacrificed, and the co-localization AAV9-TIE-shRNA (cGAS) (containing ZsGreen fluorescent protein) with the ECs of the SFO was detected to identify microinjection sites (see Additional file 1: Fig. S5A). The total volume of microinjection was 0.1 μL, consistent with our previous study [70]. The AAV9-TIE-shRNA (cGAS) was transferred to SFO and delivered by pressure via glass micropipette to the injection site for 10–15 min. The efficiency of endothelial cGAS KD in the SFO was tested using immunoblotting, and double-immunofluorescent staining of the SFO, respectively (see Additional file 1: Fig. S5B–E).

### Double-immunofluorescent staining and imaging

After the mice were anesthetized as described above, the left ventricle was perfused with 200 mL of 0.01 M PBS (pH 7.4) or 200 mL of freshly prepared 4% paraformaldehyde, and 0.1 M PB, respectively. The SFO discs were collected, fixed for 4 h after collection, placed in 20% or 30% sucrose, and dehydrated overnight at 4 °C. 30-μm-thick floating coronal sections containing the SFO were cut using a cryostat (Microm, Germany). The SFO coronal sections were washed with PBS and incubated with 0.3% Triton X-100 for 30 min, followed by incubation with 5% horse serum for 1 h at 37 °C to block non-specific proteins. Sections were incubated with primary antibodies at 4 °C. Use secondary antiserum as the secondary antibody. Fluorescent signals were observed using a Fluorview FV300 laser scanning confocal microscope (Olympus, Tokyo, Japan).

### Co-localization analysis in double-immunofluorescent image

These images were processed using ImageJ software and filtered to improve image focus. These foci were automatically segmented using the ImageJ Just Another Colocalization Plugin (JACoP) plugin for boundary segmentation and pixel-wise colocalization of the segmentation points of both channels. The Pearson coefficient was calculated using JACoP. The Pearson coefficient represents the fraction of a thresholded pixel in the green channel that is occupied by the corresponding thresholded pixel in the red channel. Statistical analysis of channel overlaps data using jmp12 analysis of variance software.

### Immunoblotting and immunoprecipitation

The SFO tissue from each mouse and cells from each group was homogenized with 1% NP40, and 1 mM PMSF in lysis buffer. In brief, protein samples (20 µg each) were subjected to SDS/PAGE on 8–12% gradient gels (Invitrogen, Carlsbad, CA, USA) and transferred to PVDF membranes. Then incubated with primary antibodies and horseradish peroxidase-conjugated secondary antibodies. Immunostaining bands can be confirmed with ECL detection reagent (WBKLS0050; Millipore). β-Actin and COX IV were used as loading controls for tissue/cell and mitochondrial proteins, respectively, to normalize the data. For immunoprecipitation, wash cell lysates with 1 µg normal rabbit IgG and 20 µL protein A + G agarose beads for 2 h at 4 °C. After centrifugation at 1000×*g* for 5 min, transfer the supernatant to a new tube and incubate with 40 μL Protein A + G Sepharose beads. After overnight incubation at 4 °C with anti-DHODH and anti-cGAS, beads were centrifuged at 12,000×*g* for 15 min at 4 °C. The precipitates were washed with RIPA and PBS, re-suspended in SDS loading buffer, and immunoblotting was performed via standard protocols.

### Cell culture

b.End 3 mouse brain endothelial cell line was purchased from the Shanghai Cell Bank of the Type Culture Collection Committee of the Chinese Academy of Sciences. Cells were grown in DMEM (Gibco, Grand Island, NY, USA) supplemented with 10% fetal bovine serum (Gibco, Grand Island, NY, USA) at 37 °C in 5% CO_2_.

### Intracellular and mitochondrial ROS detection in cultured ECs in vitro

Intracellular and mitochondrial ROS were measured according to the instructions of DCFH-DA and Mito-SOX probes. In brief, treated cultured cells were washed and incubated with 1 μM DCFH-DA and 0.5 μM Mito-SOX, respectively. After the reaction, the cells were washed, and the detection of ROS was observed under an inverted fluorescence microscope. The background was subtracted using samples without the Mito-SOX Red reagent. The mean fluorescence intensity was determined and all samples were normalized to control samples.

### Cytosolic free dsDNA detection in ECs in vitro

In brief, the cell homogenates from each group were centrifuged for 10 min and then 3 times at 980×*g* for 5 min each. Cytoplasmic supernatants were collected and centrifuged at 17,000×*g* for 25 min to obtain a cytosolic fraction free of mitochondrial and nuclear debris, and dsDNA was detected using the EZQuant dsDNA Quantification Kit. Besides, anti-TOM20 and anti-dsDNA co-stained in each group of cells by double-immunofluorescent staining was conducted. The amount of dsDNA in the cytoplasm that did not co-localize with TOM20-positive mitochondria was identified as the cytosolic free dsDNA.

### Renal sympathetic nerve activity (RSNA) records

In brief, mice were anesthetized by intraperitoneal injection of a mixture of polyurethane and sucralose at a dose of 7 mL/kg. The sympathetic bundle of the left kidney was isolated through the mouse’s left retroperitoneal incision and attached to it using a pair of platinum–iridium electrodes. The assembly of nerve and electrode was covered with silicone (Kwik-Sil, WPI, Sarasota, FL). RSNA signals were amplified (1000×) and filtered (bandwidth 30–3000 Hz) by a Grass P55C preamplifier before being input into a PowerLab data acquisition system (AD Instruments, Australia). LabChart 7 software was utilized to monitor, record, and save the signal on the computer.

### Hemodynamic measurements (echocardiography, heart rate variability, blood pressure)

Mice were anesthetized intraperitoneally with polyurethane and sucralose at a dose of 7 mL/kg. Diastolic and systolic cardiac function was assessed using transthoracic echocardiography before killing. Images were processed at 21 MHz using an MS-250 transducer driven by a Vevo 2100 color Doppler ultrasound scanner. The left ventricle was detected under the long-axis M-mode when the heart rate was approximately 400 bpm. After measurements of left ventricular end-diastolic diameter (LVEDd) and left ventricular end-systolic diameter (LVESd), the ejection fraction (EF) and fraction shortening (FS) were calculated automatically using the Teichholz method. The limb lead was made for ECG recording. Heart rate variability (HRV) analysis is also derived from the analysis module in PowerLab data acquisition system (AD Instruments, Australia). Mice blood pressure was recorded directly through an arterial cannula.

### ELISA

ELISA analysis was performed according to the manufacturer’s instructions. The plasmic NE level in each group of mice was measured using an NE ELISA kit (NOU39-K010, Eagle Biosciences). The plasmic NT-proBNP level in mice was measured using a Mouse NT-proBNP ELISA Kit (E-EL-M0834c, Elabscience). The pro-inflammatory cytokines’ level in the cellular supernatant was measured by TNF-α ELISA Kit (ab208348, Abcam) and IL-1β Mouse ELISA Kit (BMS6002, Thermo).

### Pathohistological evaluation of the myocardium

5-μm-thick slices of the myocardium were stained with Masson’s Trichrome and WGA-conjugated Alexa Fluor 488. Myocardial fibrosis was assessed with Masson’s trichrome-stained sections. In brief, red, green, and blue images of Masson’s trichrome sections were thresholded by two independent observers, and fibrosis was determined across regions of myocardial tissue using ImageJ software. Cardiomyocyte hypertrophy was assessed by measuring cross-section areas in WGA-stained sections as described previously in our published paper [71].

### Lung wet/dry ratio evaluation

The lungs were removed on both sides of each group of mice, the total weight of the lungs was measured, and then the upper part of the left lung was incised with a sharp blade, and the wet weight was measured immediately. After the dry matter was stabilized, it was dried in an oven at 60 °C for 48 h. The lung wet/dry ratio was calculated as an index for evaluating pulmonary congestion.

### Statistical analysis

Experimental data are expressed as mean ± SEM (standard error of the mean). Student’s unpaired *t*-test was used for experiments with two sample groups. For multiple group comparisons, differences between groups were determined using one-way or two-way repeated measures ANOVA. If ANOVA was significant, differences between groups were tested retrospectively using the least significant difference (LSD) test. *P* < 0.05 means the difference is statistically significant. Statistical data were analyzed using GraphPad Prism 5 software.

## Results

### C-Mito were functional compromised in HF mice

**Fig. 1:**
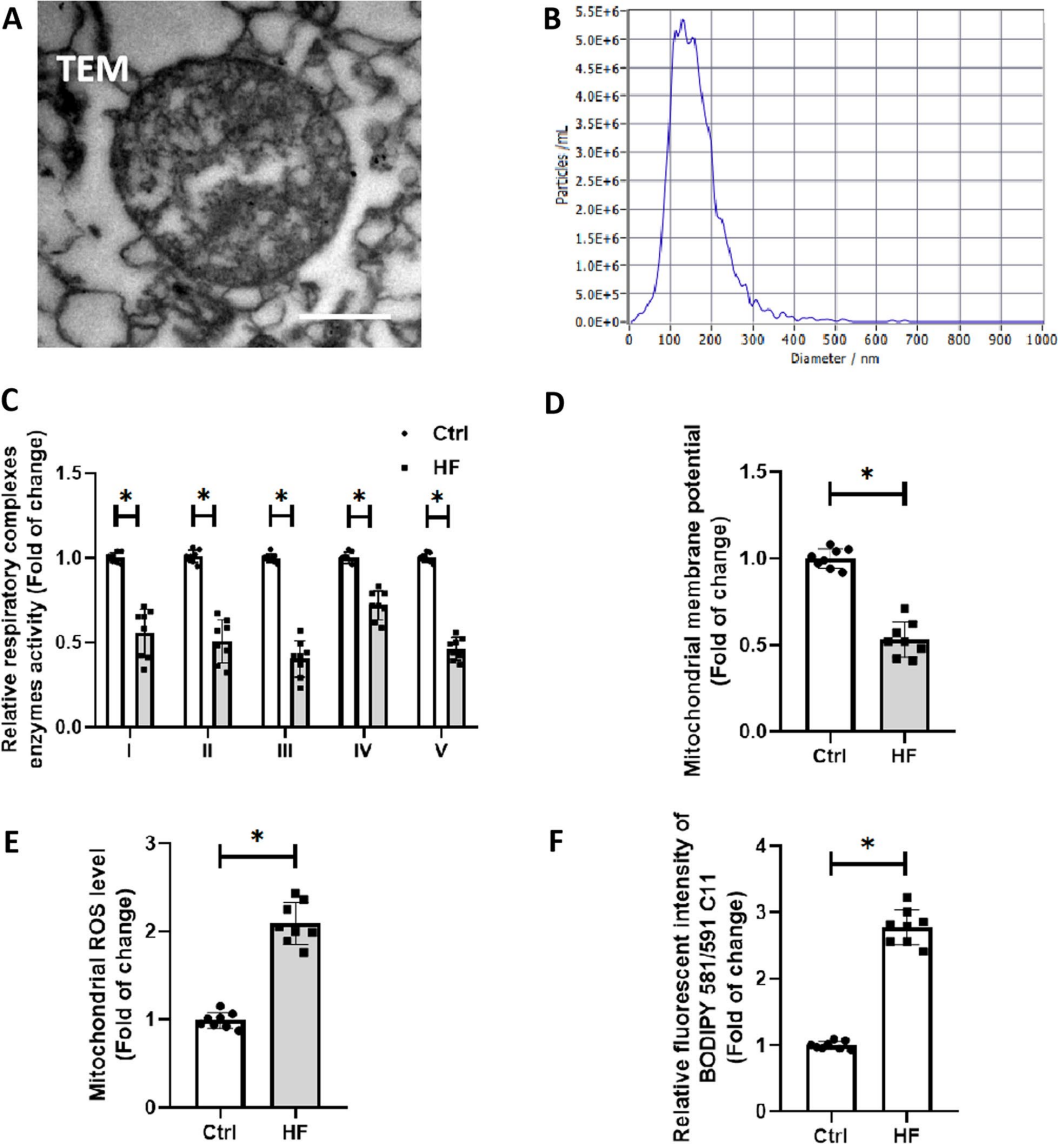
C-Mito were functionally compromised and oxidatively damaged in HF mice. **A** Representative photomicrographs of C-Mito under transmission electron microscopy. Scale bar = 50 nm. **B** Nanoparticle tracking analysis of C-Mito. **C** Respiratory complexes activity assays were performed to evaluate the function of respiratory chain of C-Mito. **D** JC-1 probe was used to detect the membrane potential of C-Mito. **E** Mito-SOX probe was used to detect the ROS level of C-Mito. **F** BODIPY 581/591 probe was used to evaluate the lipid peroxides level of C-Mito. *n* = 8, *P* < 0.05, *t* test.

We first isolated and evaluated the C-Mito from mice. TEM detected that the C-Mito retained the complete double-membrane and inner crista structure of mitochondria (Fig. 1A). The results of NTA showed that 94.3% of C-Mito had a particle size between 100 and 300 nm (Fig. 1B), which was consistent with previous reports [48, 50, 51, 66]. However, the C-Mito from HF mice presented a significant decrease of respiratory complexes activity (Fig. 1C) with a lower mitochondria membrane potential (Fig. 1D), compared to the C-Mito from control mice. Moreover, the C-Mito from HF mice contained a high level of ROS and lipid peroxides production, determined by the Mito-SOX probe (Fig. 1E) and the BODIPY 581/591 probe (Fig. 1F). These results suggested that, compared with normal intercellular mitochondria, the ROS level of C-Mito from HF mice was increased, and the function was damaged.

### C-Mito entered the ECs of the SFO in HF mice

**Fig. 2:**
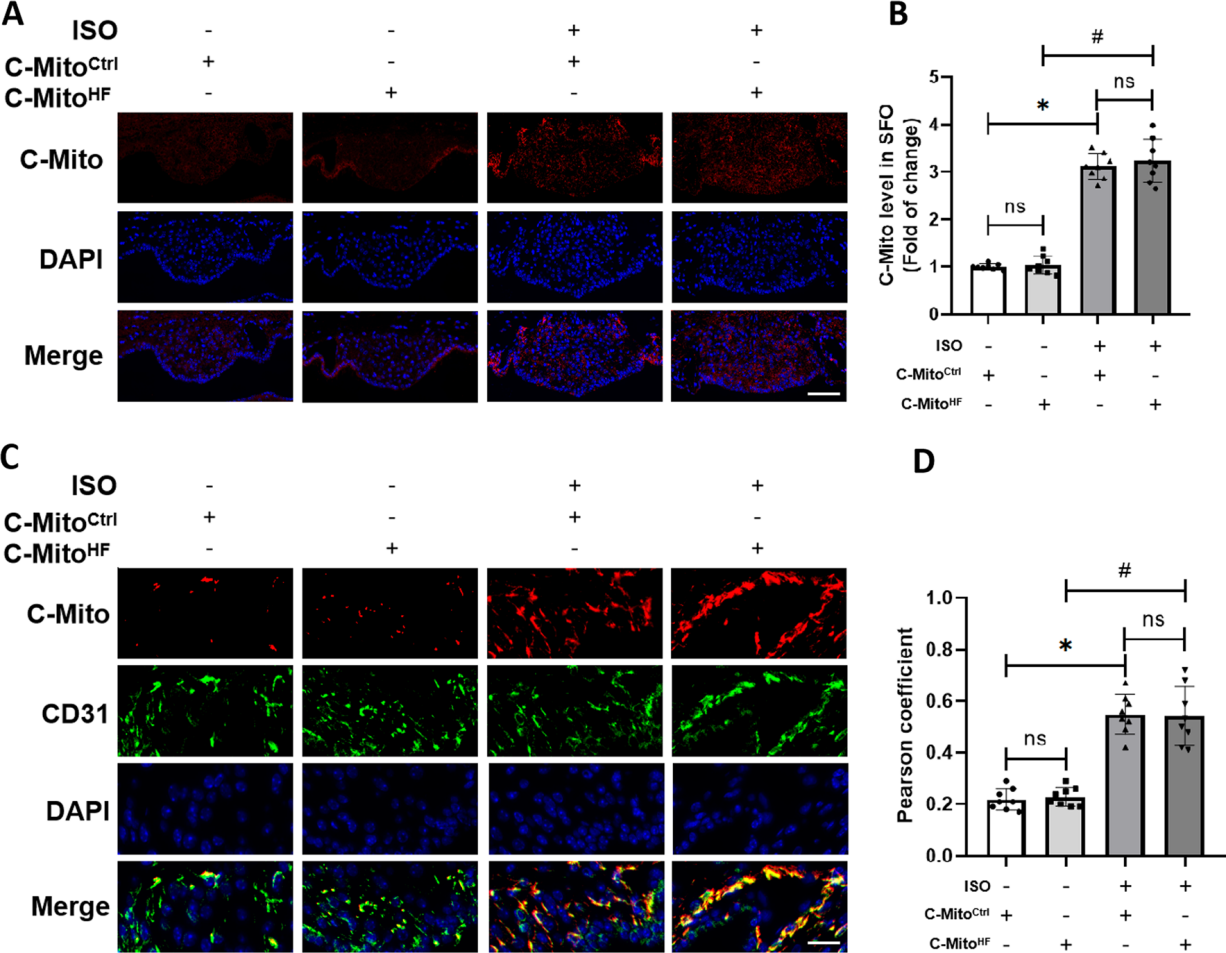
C-Mito entered ECs in the SFO of HF mice. **A** Fluorescent tracing of pre-stained C-Mito showed C-Mito were significantly enriched in the SFO. Scale bar = 100 μm. **B** Analysis showed the number of C-Mito accumulated in the SFO of HF mice was significantly higher than that in the SFO of control mice, while the number of C-Mito^HF^ entering the SFO was similar to that of C-Mito^Ctrl^. **C** Immunofluorescent staining showed C-Mito were mainly entered the ECs of the SFO in HF mice. Scale bar = 50 μm. **D** Analysis showed that the number of C-Mito^HF^ entering the ECs of the SFO was similar to that of C-Mito^Ctrl^. *n* = 8, *P* < 0.05, ANOVA LSD test

The C-Mito were pre-labeled with Mito-Tracker Red before intravenously injected into mice. The results of fluorescent staining revealed that C-Mito exhibited a notable enrichment in the brain of HF mice, compared with the control mice; and this enrichment mainly within the SFO (Fig. 2A, B). The number of C-Mito^HF^ entering the SFO was similar to that of C-Mito^Ctrl^ both in HF mice and control mice (Fig. 2A, B). Double-immunofluorescent staining was conducted to determine the exact cellular location of C-Mito in the SFO. The results showed that C-Mito were mainly entered the ECs (CD31 labeled) of the SFO in HF mice (Fig. 2C, D), instead of neurons (NeuN labeled), astroglia (GFAP labeled), and microglia (Iba-1 labeled) (Additional file 1: Fig. S2A–F). These results suggested that C-Mito entered the ECs of the SFO under HF condition.

### C-Mito^HF^ exacerbated endothelial cGAS activation in the SFO of HF mice

**Fig. 3:**
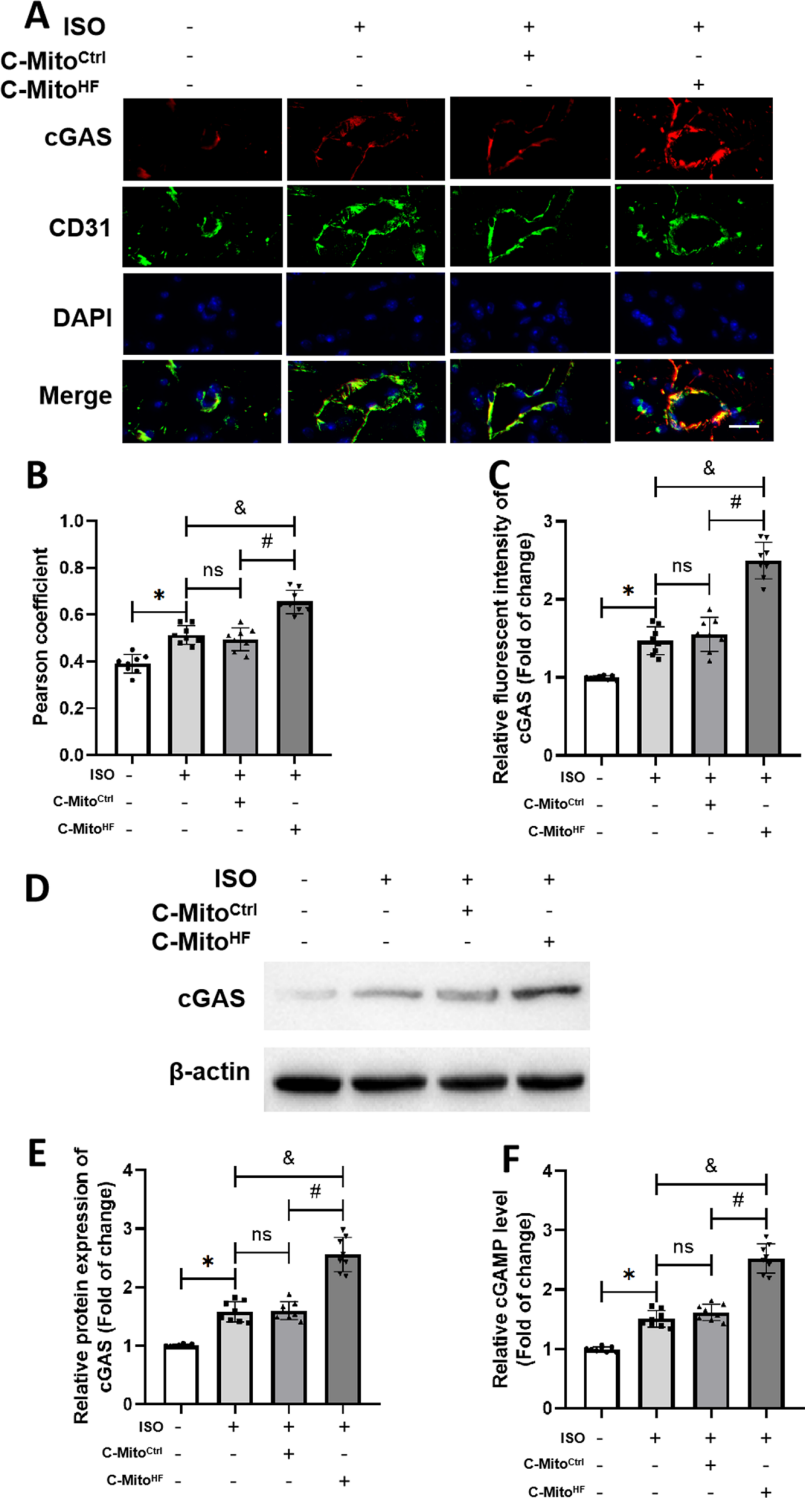
C-Mito^HF^ exacerbated endothelial cGAS activation in the SFO of HF mice. **A** Double-immunofluorescent staining of cGAS and endothelial marker CD31 in the SFO of mice. Scale bar = 50 μm. **B** Pearson coefficient analysis showed cGAS was mainly expressed in ECs of the SFO. **C** Relative fluorescent intensity analysis of cGAS showed that C-Mito^HF^ significantly exacerbated the increase of endothelial cGAS in the SFO of HF mice compared with C-Mito^Ctrl^. **D** Immunoblotting of cGAS to detect its expression in the SFO. **E** Analysis showed that C-Mito^HF^ significantly exacerbated the increase of endothelial cGAS in the SFO of HF mice compared with C-Mito^Ctrl^. **F** cGAMP level was measured in the SFO of mice. *n* = 8, *P* < 0.05, ANOVA LSD test

cGAS serves as a crucial sensor for damaged mitochondria, playing a key role in starting and intensifying neuroinflammation. Building upon this, the double-immunofluorescent staining was used to determine the expression of cGAS in the SFO. The results indicated that cGAS was mainly expressed in the ECs of the SFO (Fig. 3A, B), instead of neurons, astroglia, and microglia (Additional file 1: Fig. S3A–F). And C-Mito^HF^, other than C-Mito^Ctrl^, noticeably upregulated the expression of endothelial cGAS in the SFO of HF mice, demonstrated by immunofluorescent staining (Fig. 3A, C) and western blot results (Fig. 3D, E). Further analysis showed that compared with C-Mito^Ctrl^, C-Mito^HF^ significantly increased the level of cGAMP, the product of activated cGAS, in the SFO of HF mice (Fig. 3F). These results suggested that C-Mito^HF^ intensified both the expression and activation of endothelial cGAS in the SFO of HF mice.

### C-Mito^HF^ induced oxidative stress, cytosolic free dsDNA release, subsequent cGAS activation and inflammatory response in brain ECs

**Fig. 4:**
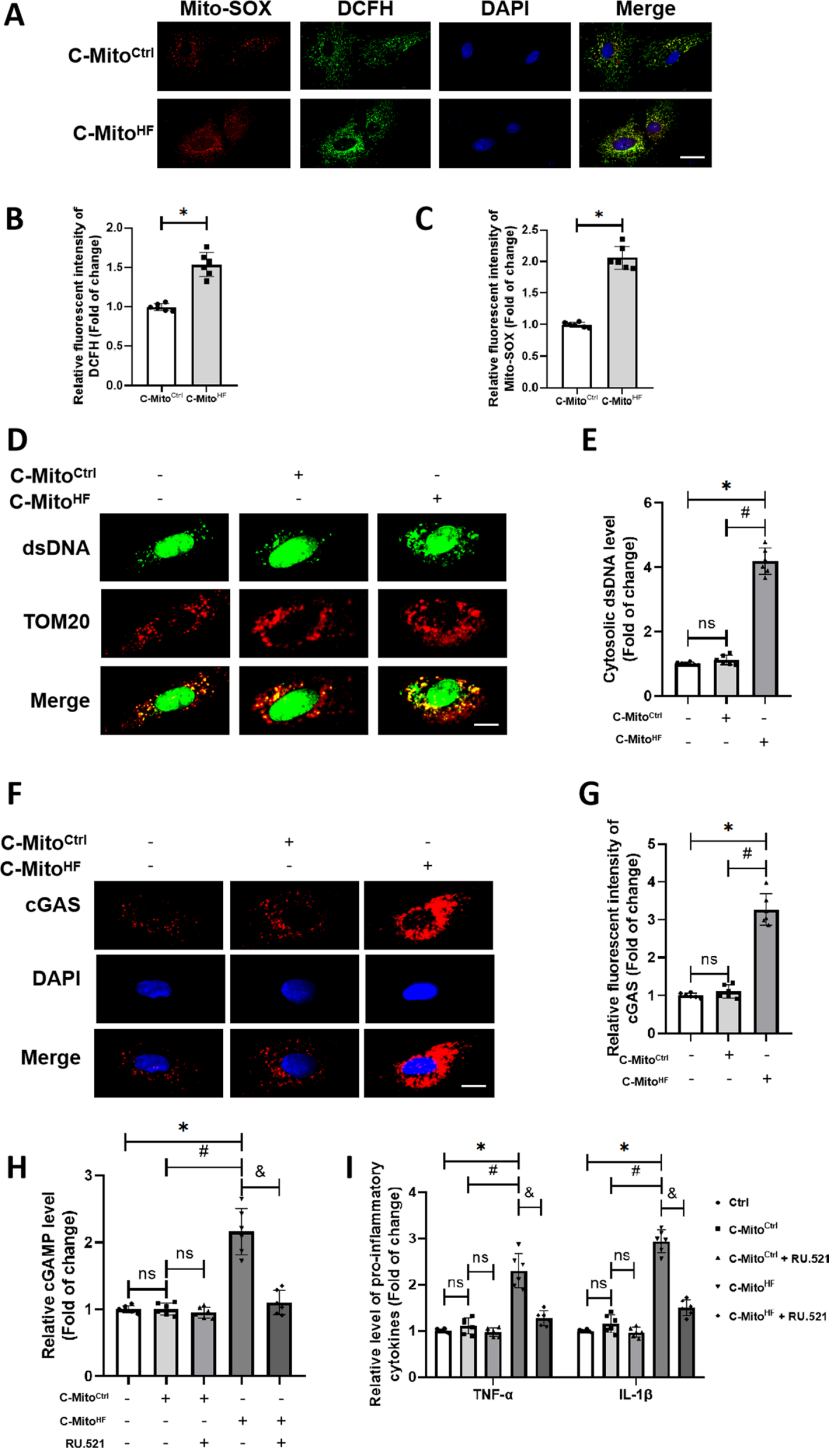
C-Mito^HF^ induced oxidative stress, cytosolic free dsDNA release, subsequent cGAS activation and inflammatory response in brain ECs. **A** DCFH-DA probe and Mito-SOX probe were used to detect ROS level in bEnd.3 cells. Scale bar = 5 μm. **B** Analysis showed that C-Mito^HF^ significantly increased intracellular ROS level compared with C-Mito^Ctrl^ in bEnd.3 cells. *n* = 6,* t* test. **C** Analysis showed that C-Mito^HF^ significantly increased mitochondria-derived ROS level compared with C-Mito^Ctrl^ in bEnd.3 cells. *n* = 6,* t* test. **D** Cytosolic free dsDNA was detected by double-immunofluorescent staining with dsDNA antibody and TOM20 antibody. Scale bar = 5 μm. **E** Cytosolic free dsDNA level was evaluated. **F** cGAS expression in C-Mito^HF^-treated bEnd.3 cells in vitro was evaluated by immunofluorescent staining. Scale bar = 5 μm. **G** Relative fluorescent intensity of cGAS in C-Mito^HF^-treated bEnd.3 cells was analyzed. **H** cGAMP level in C-Mito^HF^-treated bEnd.3 cells was measured. **I** Pro-inflammatory cytokines (TNF-α and IL-1β) production were measured in C-Mito^HF^-treated bEnd.3 cells. *n* = 6, *P* < 0.05, ANOVA LSD test

To confirm the above findings in vitro, we introduced C-Mito into the culture medium of brain endothelial cells bEnd.3 to confirm. Compared to C-Mito^Ctrl^, C-Mito^HF^ significantly promoted the cGAS expression in brain ECs (Fig. 4F, G). Using the DCFH-DA and Mito-SOX probes, we observed that C-Mito^HF^ notably heightened the production of endothelial ROS and lipid peroxides compared to C-Mito^Ctrl^ (Fig. 4A–C). In addition, C-Mito^HF^ raised the level of cytosolic free dsDNA in brain ECs compared to C-Mito^Ctrl^ (Fig. 4D, E). These results demonstrated that C-Mito^HF^-treated brain ECs experienced increased levels of both ROS and cytosolic free dsDNA, which are the pivotal triggers for cGAS activation. Furthermore, compared to C-Mito^Ctrl^, C-Mito^HF^ significantly increased cGAS level in brain ECs (Fig. 4F, G). Compared to C-Mito^Ctrl^, C-Mito^HF^ significantly elevated cGAMP production in brian ECs. When applying RU.521 to inhibit cGAS activation, the level of cGAMP decreased (Fig. 4H). By utilizing the TNF-α and IL-1β ELISA kits, we found that C-Mito^HF^ significantly enhanced pro-inflammatory cytokines production in ECs, an effect that could be attenuated by RU.521 administration (Fig. 4I). These results suggested that C-Mito^HF^ induced endothelial inflammation depending on cGAS activation.

### C-Mito^HF^ highly expressed DHODH, which interacts with cGAS to facilitate brain endothelial cGAS activation

**Fig. 5:**
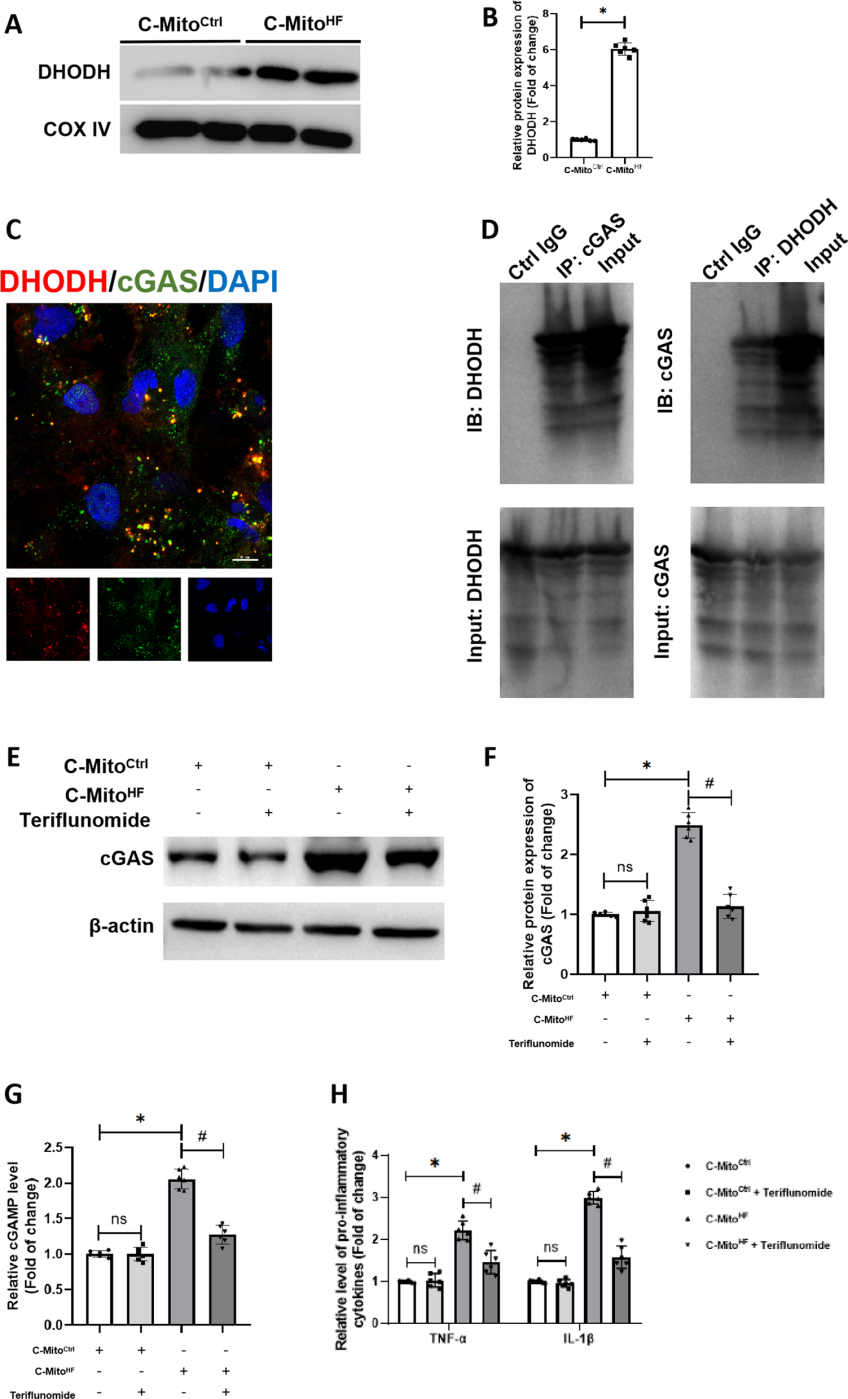
C-Mito^HF^ highly expressed DHODH, which interacts with cGAS to facilitate brain endothelial cGAS activation. **A** Immunoblotting was conducted to detect DHODH expression in C-Mito^Ctrl^ and C-Mito^HF^. **B** The results of immunoblotting showed that the expression of DHODH was higher in C-Mito^HF^ compared to C-Mito^Ctrl^.* t* test. **C** Co-localization of DHODH and cGAS was detected by double-immunofluorescent staining in bEnd.3 cells. Scale bar = 10 μm. **D** The direct interaction between DHODH and cGAS was detected by co-immunoprecipitation. **E**, **F** Immunuoblotting results showed that C-Mito^HF^ significantly increased endothelial cGAS expression, while teriflunomide pre-treatment on C-Mito^HF^ significantly mitigated the cGAS-inducing effect of C-Mito^HF^ in bEnd.3 cells. **G** Teriflunomide pre-treatment on C-Mito^HF^ significantly mitigated the cGAMP-producing effect of C-Mito^HF^ in bEnd.3 cells. **H** Pre-treated with Teriflunomide on C-Mito^HF^ ameliorated the pro-inflammatory effect of C-Mito^HF^ in bEnd.3 cells. *n* = 6, *P* < 0.05, ANOVA LSD test

Interestingly, we found that the expression of DHODH, a key regulator of mitochondrial redox balance, was significantly increased in C-Mito^HF^ compared to C-Mito^Ctrl^ (Fig. 5A, B). The co-localization of DHODH and cGAS was observed in brain ECs (Fig. 5C) and the direct interaction between DHODH cGAS was confirmed by co-immunoprecipitation (Fig. 5D). When using the DHODH inhibitor Teriflunomide, the C-Mito^HF^-treated upregulation of cGAS in brain ECs was suppressed (Fig. 5E, F). The same results were observed in the ECs of the SFO in HF mice through immunofluorescent staining (Additional file 1: Fig. S4A, B). Further analysis showed that pre-treated with Teriflunomide on C-Mito^HF^ ameliorated cGAMP expression (Fig. 5G) as well as TNF-α and IL-1β production (Fig. 5H). These results suggested that C-Mito^HF^ highly expressed DHODH, which interacted with cGAS. Inhibition of DHODH mitigated the C-Mito^HF^-induced cGAS activation and further cellular inflammation in brain ECs.

### C-Mito^HF^ promoted neuroinflammation and neuronal sensitization in the SFO depending on endothelial cGAS in HF mice

**Fig. 6:**
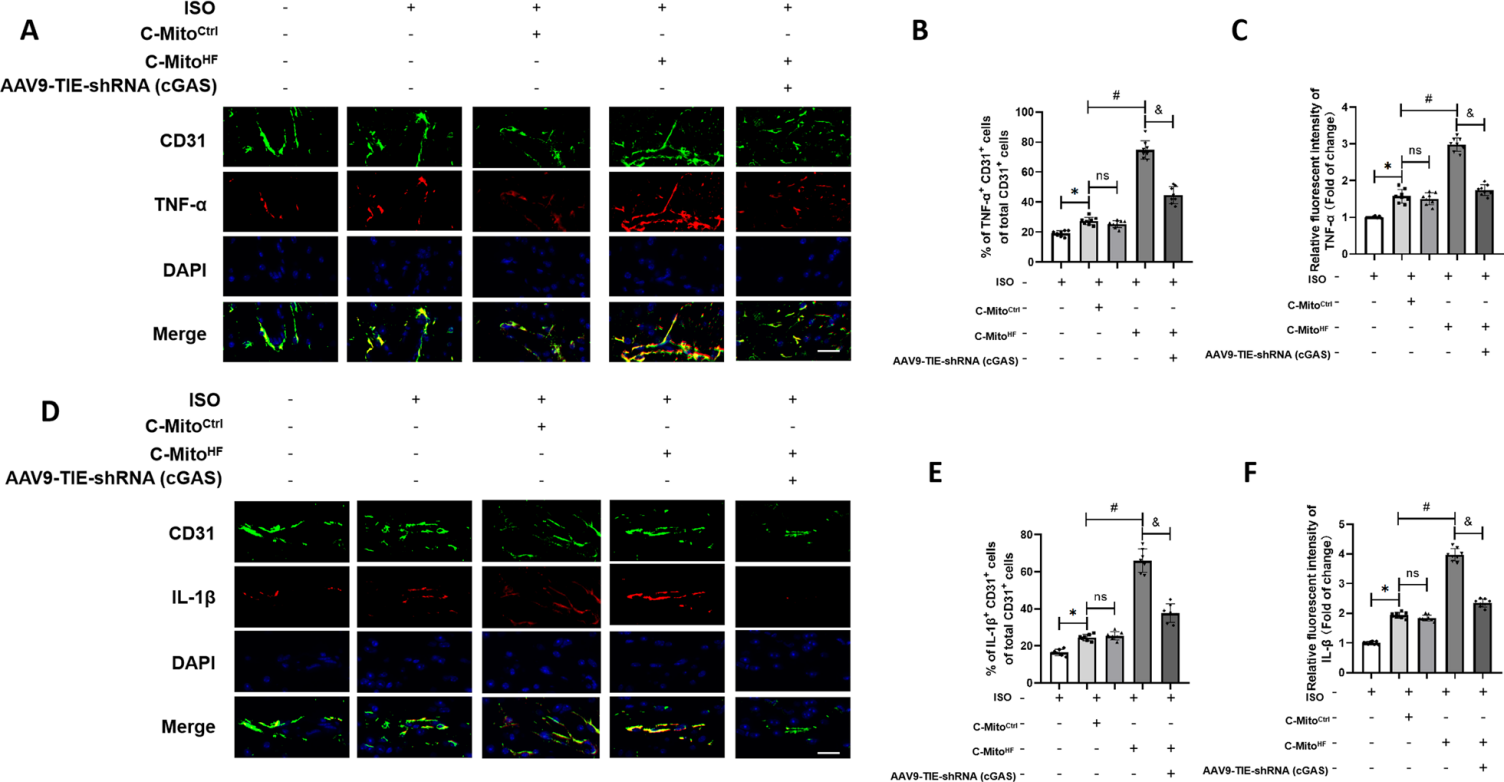
C-Mito^HF^ promoted cGAS-dependent pro-inflammatory cytokines production in ECs of the SFO in HF mice. **A**–**C** Double-immunofluorescent staining showed that compared with C-Mito^Ctrl^, C-Mito^HF^ exacerbated endothelial expression of pro-inflammatory cytokines TNF-α in the SFO of HF mice, which could be mitigated by endothelial cGAS KD in the SFO. Scale bar = 100 μm. **D**–**F** Double-immunofluorescent staining showed that compared with C-Mito^Ctrl^, C-Mito^HF^ exacerbated endothelial expression of pro-inflammatory cytokines IL-1β in the SFO of HF mice, which could be mitigated by endothelial cGAS KD in the SFO. Scale bar = 100 μm. *n* = 8, *P* < 0.05, ANOVA LSD test

**Fig. 7:**
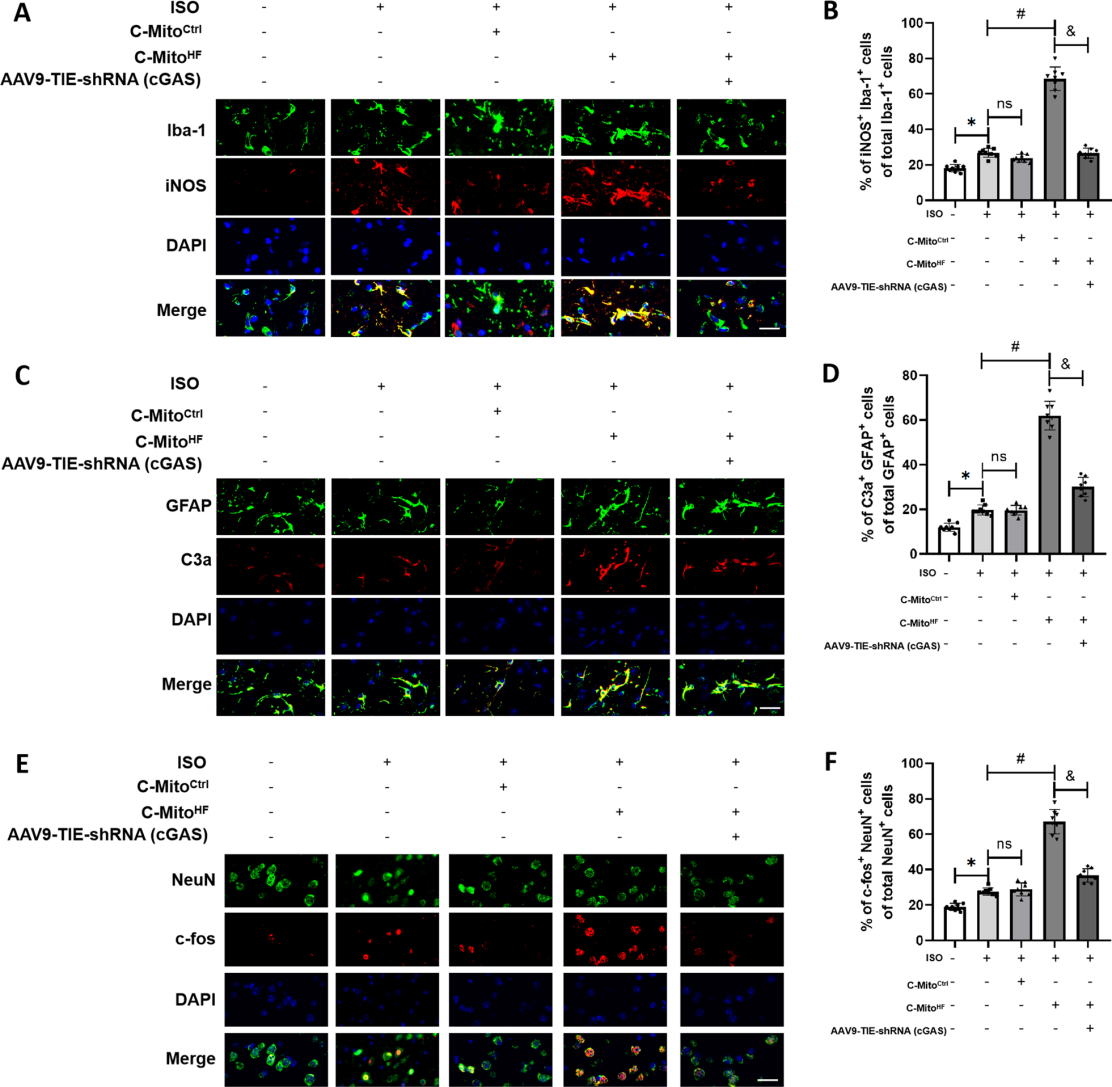
C-Mito^HF^ promoted glial activation and neuronal sensitization in the SFO depended on endothelial cGAS in HF mice. **A**, **B** Double-immunofluorescent staining showed that compared with C-Mito^Ctrl^, C-Mito^HF^ exacerbated microglial M1 polarization in the SFO of HF mice, which could be mitigated by endothelial cGAS KD in the SFO. Scale bar = 100 μm. **C**, **D** Double-immunofluorescent staining showed that compared with C-Mito^Ctrl^, C-Mito^HF^ exacerbated astroglial A1 transition in the SFO of HF mice, which could be mitigated by endothelial cGAS KD in the SFO. Scale bar = 100 μm. **E**, **F** Double-immunofluorescent staining showed that compared with C-Mito^Ctrl^, C-Mito^HF^ exacerbated neuronal sensitization in the SFO of HF mice, which could be mitigated by endothelial cGAS KD in the SFO. Scale bar = 100 μm. *n* = 8, *P* < 0.05, ANOVA LSD test

To confirm if the focused presence of cGAS in ECs within the SFO is a critical site connecting mitochondrial damage to the onset of neuroinflammatory processes in vivo, we constructed specific endothelial cGAS knocking down by injecting AAV9-TIE-shRNA (cGAS) into the SFO of mice (Additional file 1: Fig. S5A–C). Neither the SFO-specific knockout of endothelial cGAS by injection of AAV9-TIE-shRNA (cGAS) into the SFO of control mice nor the empty virus vector promoted the SFO neuroinflammation and sympathetic activation (Additional file 1: Fig. S5D, E). The immunofluorescent staining results showed that compared to C-Mito^Ctrl^, C-Mito^HF^ exacerbated endothelial expression of pro-inflammatory cytokines TNF-α (Fig. 6A–C) and IL-1β (Fig. 6D–F) in the SFO of HF mice, which could be attenuated by cGAS KD. These findings were consistent with the in vitro results. When applying the neuroinflammation indicators, we observed that C-Mito^HF^ significantly increased the iNOS expression in microglia (Fig. 7A, B) as well as the C1a expression in astroglia (Fig. 7C, D) of the SFO in HF mice, compared with C-Mito^Ctrl^. These results suggested that C-Mito^HF^ exacerbated glial pro-inflammatory transition in the SFO of HF mice depending on endothelial cGAS. Furthermore, the results of immunofluorescent staining showed that compared to C-Mito^Ctrl^, C-Mito^HF^ significantly increased the expression of c-fos (the marker of neuronal sensitization) in the neurons of the SFO in HF mice (Fig. 7E, F). All these results suggested that C-Mito^HF^ intensified neuroinflammation and neuronal sensitization depending on endothelial cGAS in HF mice.

### C-Mito^HF^ aggravated sympathetic hyperactivation and myocardial sympathetic hyperinnervation depended on the SFO endothelial cGAS in HF mice

**Fig. 8:**
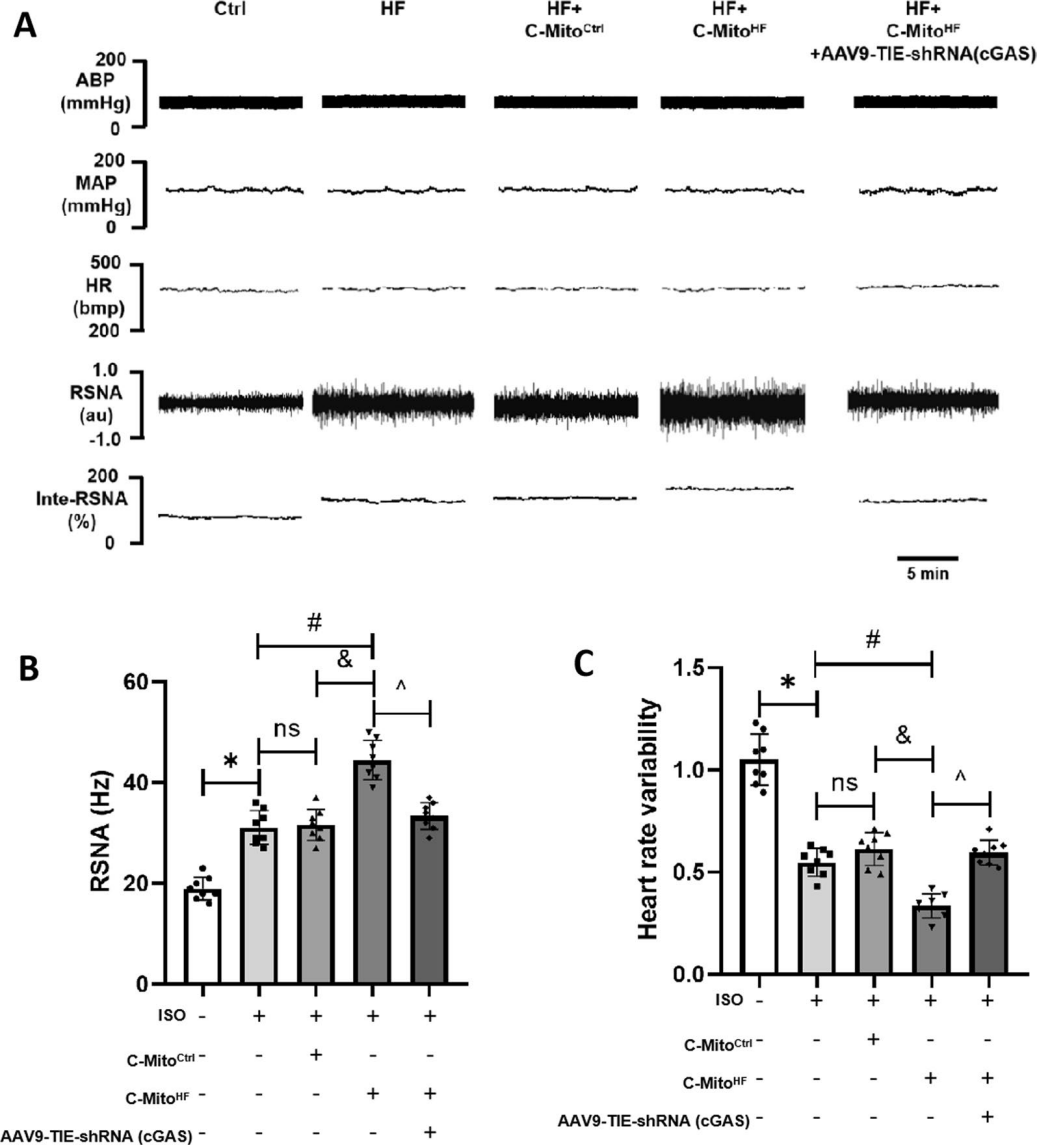
C-Mito^HF^ increased cardiac sympathetic hyperactivation depending on the SFO endothelial cGAS in HF mice. **A** Renal sympathetic activity (RSNA) of mice were evaluated to determine the cardiovascular sympathetic output in C-Mito^HF^-treated HF mice. **B** Compared to C-Mito^Ctrl^, C-Mito^HF^ significantly increased the RSNA level in HF mice, while endothelial cGAS KD in the SFO ameliorated the sympathoexcitation effect of C-Mito^HF^ in HF mice. **C** The results of heart rate variability (HRV) analysis showed C-Mito^HF^ decreased LF/HF ratio of HF mice, which implies cardiac sympathetic/parasympathetic tones imbalance with the sympathetic overactivation, while endothelial cGAS KD in the SFO ameliorated this effect. *n* = 8, *P* < 0.05, ANOVA LSD test

**Fig. 9:**
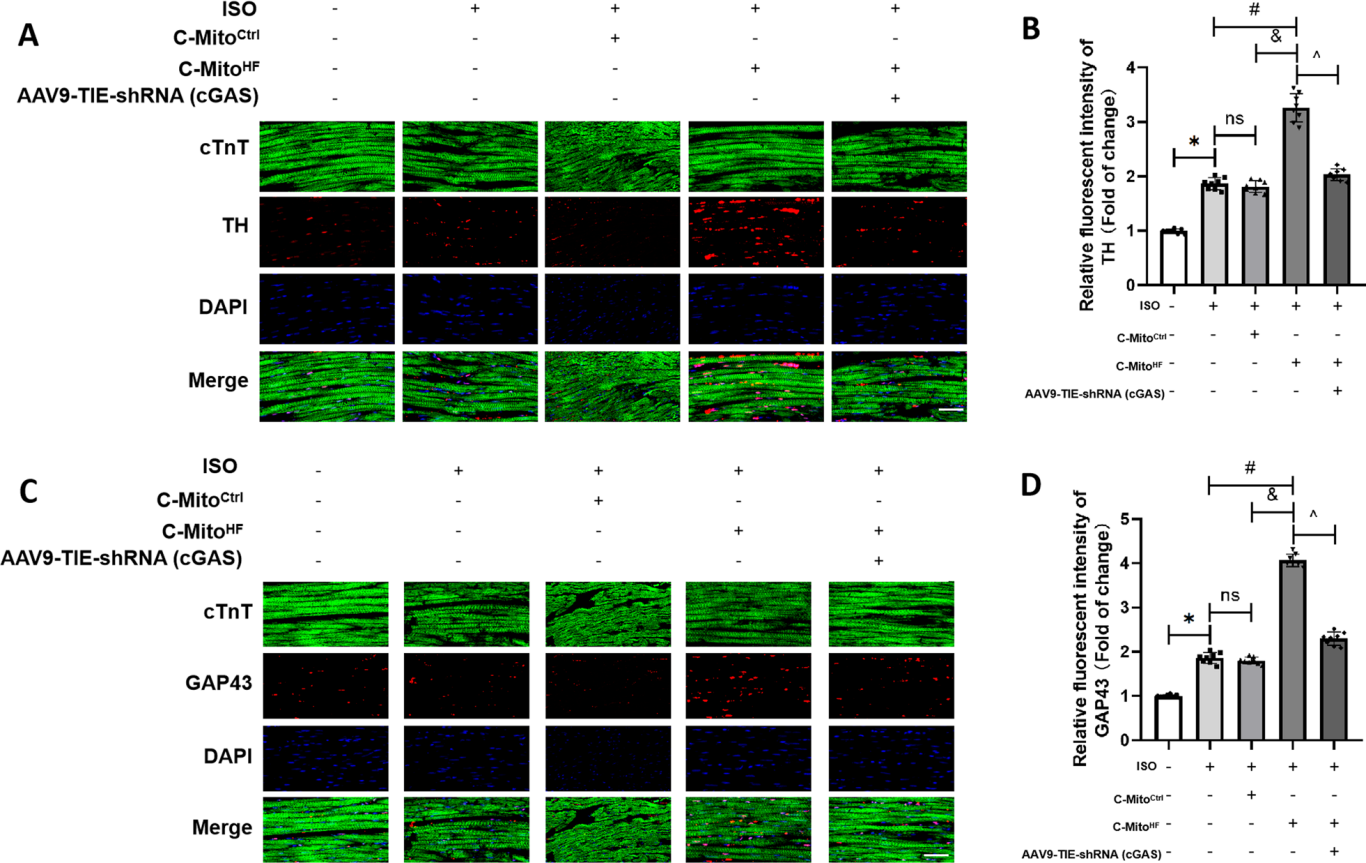
C-Mito^HF^ aggravated myocardial sympathetic hyperinnervation depended on the SFO endothelial cGAS in HF mice. Immunofluorescent staining results showed that, compared to C-Mito^Ctrl^, C-Mito^HF^ exacerbated sympathetic hyperinnervation in the myocardium of HF mice, shown by increased TH-positive nerve fibers (**A**, **B**) and GAP43 positive sprouting nerves (**C**, **D**), which could be ameliorated by endothelial cGAS KD in the SFO of HF mice. Scale bar = 150 μm. *n* = 8, *P* < 0.05, ANOVA LSD test

Given the contributions of neuroinflammation to the sympathetic activity in cardiovascular diseases, we measured the renal sympathetic nerve activity (RSNA) of mice subjected to different C-Mito treatments. The results revealed that, in comparison to C-Mito^Ctrl^, C-Mito^HF^ significantly raised the RSNA level in HF mice, an effect that was ameliorated by suppressing endothelial cGAS in the SFO (Fig. 8A, B). The analysis of heart rate variability demonstrated that C-Mito^HF^ enhanced cardiac sympathetic output in HF mice, which could be alleviated by inhibiting the cGAS expression in the ECs of the SFO (Fig. 8C). In addition, in contrast to C-Mito^Ctrl^, C-Mito^HF^ elevated plasmic norepinephrine (NE) levels in HF mice, while endothelial cGAS knockdown in the SFO mitigated this impact (Additional file 1: Fig. S6). These results demonstrated that C-Mito^HF^ aggravated sympathetic hyperactivation of HF mice via endothelial cGAS activation in the SFO. Furthermore, we detected the myocardial sympathetic innervation by conducting immunofluorescent staining of TH (a marker of sympathetic nerves) and GAP43 (a marker of newborn nerves). The results indicated that compared to C-Mito^Ctrl^, C-Mito^HF^ increased the number of TH-positive nerve fibers (Fig. 9A, B) and GAP43-positive sprouting nerves in the myocardium of HF mice (Fig. 9C, D), which were ameliorated by reducing endothelial cGAS in the SFO of HF mice (Fig. 9A–D). These results suggested that C-Mito^HF^ aggravated sympathetic hyperactivation and myocardial sympathetic hyperinnervation via endothelial cGAS-regulated neuroinflammation in HF mice.

### C-Mito^HF^ exacerbated myocardial remodeling and systolic dysfunction in HF mice through activating endothelial cGAS in the SFO

**Fig. 10:**
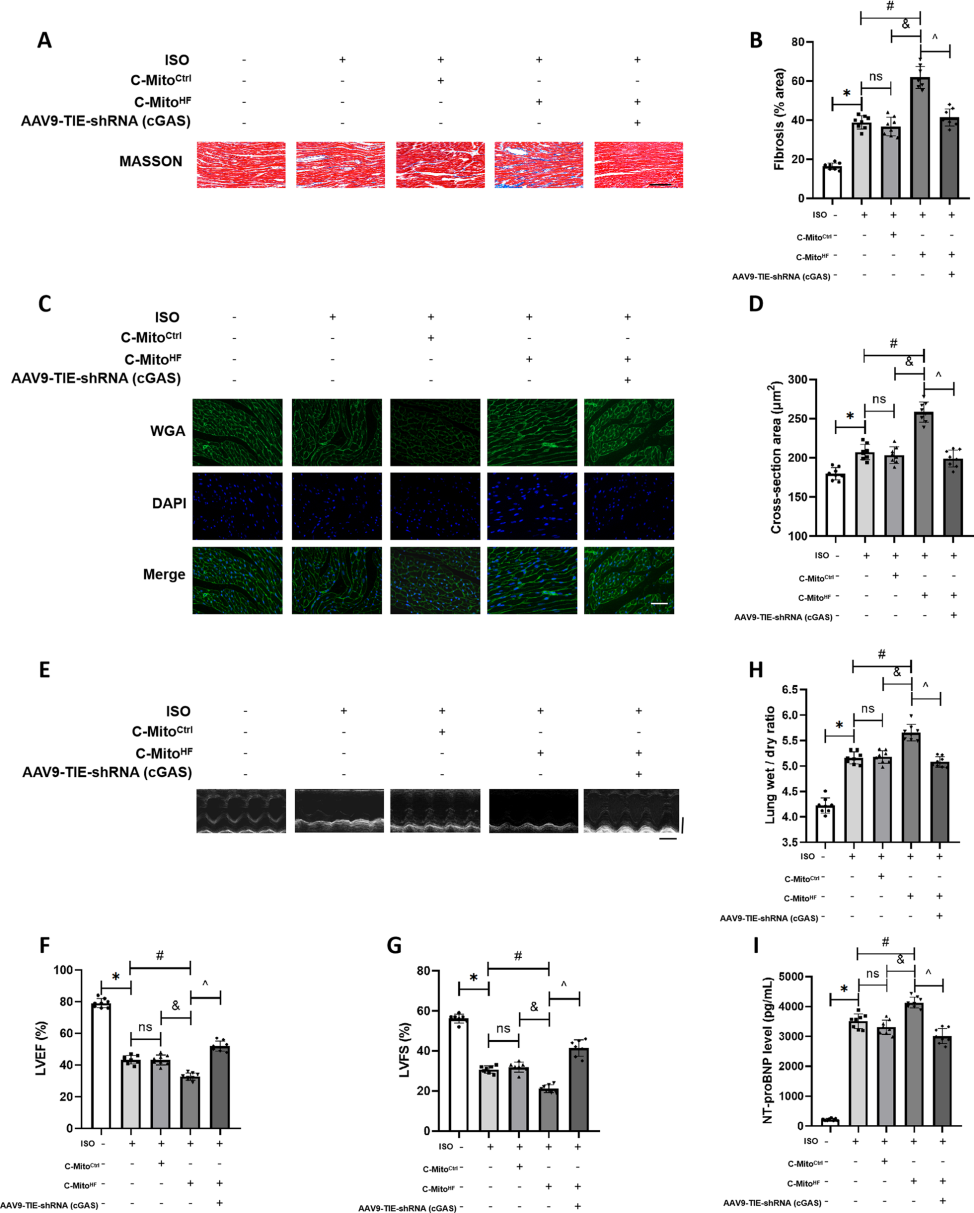
C-Mito^HF^ exacerbated myocardial remodeling and systolic dysfunction in HF mice through activating endothelial cGAS in the SFO. **A** Masson staining were performed to assess the myocardial fibrosis in C-Mito^HF^-treated HF mice. Scale bar = 150 μm. **B** Compared to C-Mito^Ctrl^, C-Mito^HF^ exacerbated interstitial and perivascular fibrosis in HF mice, which could be ameliorated by endothelial cGAS KD in the SFO. **C** WGA staining were performed to assess the cardiomyocyte hypertrophy in C-Mito^HF^-treated HF mice. Scale bar = 150 μm. **D** Compared to C-Mito^Ctrl^, C-Mito^HF^ exacerbated cardiomyocyte hypertrophy in HF mice, which could be ameliorated by endothelial cGAS KD in the SFO. **E** Echocardiography was conducted to evaluate the cardiac systolic function. Compared to C-Mito^Ctrl^, C-Mito^HF^ significantly decreased the LVEF (**F**) and LVFS (**G**) in HF mice, while endothelial cGAS KD in the SFO mitigated the left heart systolic dysfunction in mice. **H** Lung wet/dry ratio was evaluated in mice. I. Plasmic NT-proBNP level was measured in mice. *n* = 8, *P* < 0.05, ANOVA LSD test

Finally, myocardial remodeling and cardiac functions were analyzed. Compared to C-Mito^Ctrl^, C-Mito^HF^ exacerbated interstitial and perivascular fibrosis (Fig. 10A, B) and myocardial hypertrophy (Fig. 10C, D) in HF mice. Inhibiting the endothelial cGAS expression in the SFO could ameliorate these effects. Echocardiography results indicated that compared to C-Mito^Ctrl^, C-Mito^HF^ significantly reduced the left ventricular ejection fraction (LVEF) and left ventricular fraction shortening (LVFS) in HF mice. Inhibition of endothelial cGAS in the SFO improved the cardiac systolic function (Fig. 10E–G). The results of mice lung wet/dry ratio revealed that compared to C-Mito^Ctrl^, C-Mito^HF^ worsened pulmonary congestion in HF mice, but was lessened by endothelial cGAS knockdown in the SFO (Fig. 10H). Also, compared to C-Mito^Ctrl^, C-Mito^HF^ notably increased the plasmic NT-proBNP level in HF mice, which was lowered by endothelial cGAS suppression in the SFO (Fig. 10I). These results suggested that C-Mito^HF^ exacerbated myocardial remodeling and cardiac dysfunction in HF mice through activating endothelial cGAS in the SFO.

## Discussion

Mito have been identified as a novel mediator for the inter-organ communication. However, their structures and functions in HF are still unknown. In the present study, we reported for the first time C-Mito were functionally impaired with reduced respiratory complexes activity and increased oxidative stress insult in HF mice. This is consistent with previous studies on other pathological states, including brain death, cardiogenic death, and obesity [51, 65, 66]. Next, we found that C-Mito mainly entered the SFO of the brain. SFO is different from other cerebral nucleus in that it does not contain an intact blood–brain barrier. Therefore, SFO could sense inflammatory stimuli from circulation which plays a role in inter-communication between peripheral circulation and the brain. Previous studies have confirmed that alternation in blood–brain barrier permeability could occur in animal models of hypertension, hyperlipidemia, myocardial infarction, MI/R, or HF [72–78]. This change is manifested by microleakage of cerebrovascular and perivascular inflammatory infiltration. This may explain why the number of incorporated C-Mito in the SFO of HF mice was significantly higher than that of control mice.

It has been widely accepted that neuroinflammation-mediated sympathetic regulatory center dysfunction is involved in sympathetic hyperactivation in cardiovascular diseases [79–81]. Our previous studies revealed that neuroinflammation induced by microglia activation is involved in the increase of sympathetic output [70, 71, 82]. Since we found that C-Mito^HF^ promoted endothelial cGAS activation, endothelial inflammatory response in the SFO of HF mice was evaluated. This suggests that C-Mito may transmit ROS and inflammatory signals in the inter-organ communication of HF. Similarly, a report has also confirmed that damaged C-Mito released by adipocytes in obese mice induced by high-fat diet could carry a large amount of ROS. The incorporation of oxidatively damaged C-Mito significantly induces the activation of oxidative stress preconditioning signaling in cardiomyocytes, and plays a protective role against MI/R injury [51]. The mechanism of C-Mito in the adipose–heart axis provides a mechanistic explanation for the paradox of the cardioprotective effect of obesity in patients with myocardial infarction. Previous clinical studies have shown that myocardial inflammation in patients with acute myocardial infarction is accompanied by cerebral inflammation. The occurrence of neuroinflammation is associated with poor prognosis in patients with myocardial infarction [83]. Could C-Mito involved in the dysregulation of the heart–brain axis in HF, reported for the first time in this study, be a key mediator of inflammatory information transmission between the heart and brain? We all know that HF is an important risk factor for brain dysfunction, including perioperative delirium after cardiac surgery and dementia in patients with chronic HF [84–87]. So might C-Mito be a therapeutic target for intervention in cardiovascular disease-related brain dysfunction? Whether the intervention of C-Mito could benefit both the heart and the brain in patients with myocardial injury needs to be further explored in future studies.

Our results show that the damaged C-Mito mainly enter SFO endothelial cells rather than glial cells and neurons. Then, our results showed that the entry of C-Mito^HF^ into the SFO ECs significantly activated cGAS. We found that C-Mito^HF^ significantly promoted ROS accumulation and cytosolic mtDNA release. Cytosolic free mtDNA is a potent inducer of cGAS, and ROS is a newly identified activator of cGAS. Besides, there is a bidirectional regulation between cGAS and ROS. Previous studies have shown that the cGAS signaling directly regulates the GPX4-mediated lipid peroxidation pathway [88], indicating that cGAS signaling could affect the degradation process of ROS-related peroxidation damage. In addition, cGAS is considered to be the main activator of senescence-associated secretory phenotype switch, which is characterized by the release of inflammatory cytokines accompanied by the production of a large amount of ROS [89–92]. Previous literature has shown that cGAS is not only passively sensing mitochondrial state, but actively regulates mitochondrial metabolism and dynamics as well as mitochondrial biogenesis [93, 94]. All the above mitochondrial biological processes could profoundly impact mitochondrial ROS metabolism. It is well-accepted that, mtDNA, ROS and various constituent proteins carried by mitochondria are strong inducers of pro-inflammatory signaling [52–55, 95]. We found C-Mito^HF^ in turn strongly stimulated the production of pro-inflammatory cytokines (TNF-α and IL-1β) production in ECs, and ultimately aggravated the pathological communication between ECs and glial cells/neurons, leading to neuroinflammation and subsequent cardiac sympathetic hyperactivation. The predominant expression of cGAS in ECs of the SFO highlights a specific cellular locus where cGAS activity may orchestrate key events in the intricate interplay between mitochondrial damage and the neuroinflammatory cascade.

In the present study, we surprisingly found that mitochondrial redox regulator DHODH could directly interact with cGAS, and further promote cGAS activation in ECs. DHODH is the mitochondria-localized rate-limiting enzyme for the de novo synthesis of pyrimidines, which catalyzes the conversion of dihydroorotate to orotate with quinone as an electron acceptor. DHODH is considered as an important mitochondrial redox regulator because it regulates the efficiency of the mitochondrial electron transport chain and is closely related to the electron leakage of the mitochondrial respiratory chain [96–99]. Recent study has reported that DHODH regulates cellular sensitivity to lipid peroxidation and ferroptosis [96–99]. Our study suggested that DHODH might be the new molecular target for the intervention of C-Mito^HF^. Our results suggested that Teriflunomide, a clinically used anti-rheumatic drug [100], may have clinical translation potential for the intervention of sympathetic hyperactivation in HF.

Our study demonstrates for the first time that C-Mito^HF^ aggravated myocardial remodeling and HF progression. It is generally believed that HF is not a simplified single organ failure, but a systemic disease, involving the pathological interaction of heart–brain, heart–lung, heart–liver, heart–kidney, etc. [101–108]. In addition to the neuroendocrine system involved in the coordination of multi-organ functions [109], the multi-organ communication mediated by exosomes and extracellular vesicles through circulating blood also plays an irreplaceable role [110, 111]. Besides, extracellular vesicles and exosomes have potential theranostics value, which has been paid attention to by researchers [112]. In studies of heart–brain axis dysfunction in the pathogenesis of HF, previous studies have focused on exosomes released by the cardiomyocytes, which could enter the myocardium-innervated sympathetic nerves to modulate noradrenaline releasing [12], or enter the sympathetic regulatory center, such as Rostral ventrolateral medulla (RVLM) [113]. It has been found that the exosomes released from stressed myocardium contained microRNAs that regulate mitochondrial energy metabolism and redox homeostasis of sympathetic regulating neurons. The entry of these exosomes into sympathetic neurons could induce oxidative stress resulting in increased cardiovascular sympathetic tone. Our study confirms that C-Mito^HF^ are also involved in the pathogenesis of HF and play an important role in heart–brain axis abnormalities. Whether it is involved in the pathological communication between the heart and other organs in the pathogenesis of HF is also a topic worth exploring.

As a novel discovered mediator for the inter-communication of distant organs, the specific biological information transmitted by C-Mito between donor cells and recipient cells is still poorly understood. The cellular origin of C-Mito is a very interesting topic. Studies have shown that cardiomyocytes, neurons, glial cells, and macrophages could actively release functional mitochondria into the extracellular space, and almost all cells could receive extracellular mitochondria [44, 114–119]. These findings suggest that the extracellular release of mitochondria may be a conservative mechanism for mitochondrial quality control, and C-Mito may be one of the basic mechanisms for intercellular communication for signal transduction and metabolism coordination. Stressed cardiomyocytes release damaged mitochondria into the intercellular space for resident macrophages to phagocytosis, which is a novel approach for mitochondrial quality control of myocardium. Mitochondrial turning is critical to the energy supply of the heart. Are C-Mito released by cardiomyocytes? If so, what is the proportion of C-Mito of myocardial origin? This is the question worth exploring in the future. Moreover, is the distribution and aggregation of C-Mito in various organs random? Are C-Mito of different cell origins specific in their organ distribution? Are the target cells of C-Mito random or are they also specific? These are also questions worth exploring. Our results suggest that neither the aggregation nor the cellular distribution of C-Mito in the brain is random. The exact molecular mechanism of how ECs take up C-Mito is also unclear. It is reported that macrophages mediate the endocytosis of extracellular mitochondria through the MertK receptor [44]. Is there a specific ligand–receptor interaction between the membrane proteins of C-Mito and the membrane proteins of recipient cells? Answering these questions will provide us with great value in understanding the biological role of C-Mito transmission.

Renal sympathetic nerve activity (RSNA) measurement is a widely employed method for monitoring central sympathetic output in basic cardiovascular disease research. Key sympathetic centers such as the subfornical organ (SFO), paraventricular nucleus (PVN), rostral ventrolateral medulla (RVLM), and others ultimately innervate the cardiovascular system through interneurons of the cervicothoracic spinal cord. These interneurons project sympathetic nerve endings, regulating cardiovascular activity via the thoracolumbar segment of the spinal cord. The renal sympathetic nerve is frequently chosen for recording sympathetic activity due to its ease of identification and surgical dissociation. It plays a crucial role in the cardiovascular sympathetic system and interacts with the peripheral renin–angiotensin–aldosterone system, contributing to cardiovascular pathological remodeling. RSNA detection serves as a reflection of central cardiovascular sympathetic output in animal models with cardiovascular diseases, as highlighted in our and other research groups’ publications [70, 120–122].

While stellate ganglion nerve activity (SGNA) is a direct tool for studying cardiac sympathetic nerve activity, its recording involves thoracotomy, resulting in significant trauma and complicating routine application. Subcutaneous nerve activity (SCNA) and skin sympathetic nerve activity (SKNA) were developed to address these challenges. SGNA records activity through thoracotomy, which can be traumatic. SCNA recording, achieved by embedding the electrode in the subcutaneous chest tissue, roughly reflects immediate SGNA and cardiac sympathetic nerve activity. Both SCNA and SKNA, while less precise, offer alternatives with lower trauma and may be more suitable for clinical applications than basic experiments. Previous studies have demonstrated good synchronization between RSNA and SCNA, and their activities interact to promote cardiovascular sympathetic damage [123]. While both RSNA and SGNA are crucial for directly recording cardiovascular sympathetic output in animal experiments, RSNA, not requiring thoracotomy, offers a more accessible and practical approach. Therefore, RSNA was selected as the method to detect central sympathetic output in this study.

There were several technical limitations in this study. First, because there is no established technique for C-Mito tracing, we have not been able to identify the differences in the cellular origin of C-Mito in HF mice and control mice. In the future, we plan to conduct specific fluorescent labeling of mitochondrial proteins in cardiomyocytes to trace the inter-organ migration of injured myocardial mitochondria. In addition, the specific molecular mechanism of how endothelial cells take up circulating mitochondria remains to be further elucidated.

## Conclusion

Collectively, C-Mito could incorporated into the endothelial cells of the SFO in HF mice and subsequently activated endothelial cGAS. C-Mito^HF^ were oxidatively damaged with highly expressed DHODH. DHODH from C-Mito^HF^ directly interacted with cGAS and facilitated cGAS activation in ECs. C-Mito^HF^ induced EC-derived neuroinflammation in the SFO and further aggravated myocardial sympathetic hyperactivation and pathological remodeling. Targeting C-Mito might be a new therapeutic strategy for interfering with heart–brain axis dysfunction in HF.

### Supplementary Information

**Additional file 1: Figure S1.** The expression of mitochondrial respiratory complexes (A), mitochondrial outer membrane protein TOM20, inner membrane protein TIM50, and matrix protein Hsp60 (B) were detected by immunoblotting in order to verify that the isolated microparticles were C-Mito. *n* = 8. **Figure S2.** Immunofluorescent staining was conducted to detect the co-localization of C-Mito and neurons (A, B), astroglia (C, D), and microglia (E, F). *n* = 8. ANOVA LSD test. **Figure S3.** Immunofluorescent staining was conducted to detect the co-localization of cGAS and neurons (A, B), astroglia (C, D), and microglia (E, F). *n* = 8. ANOVA LSD test. **Figure S4.** pre-treated with Terflunomide on C-Mito^HF^ mitigated the cGAS-upregulating effect of C-Mito^HF^ in ECs of the SFO in HF mice in vivo. *n* = 8, *P* < 0.05, *t* test. **Figure S5.** Verification of endothelial cGAS KD in the SFO of mice by AAV9-TIE-shRNA (cGAS) injection. A. The co-localization of AAV9 virus and ECs in the SFO of mice. Scale bar = 200 μm. B, C. Immunofluorescent staining showed successful endothelial cGAS KD in the SFO of mice. Scale bar = 100 μm. D. Neither the SFO-specific knockout of endothelial cGAS by injection of AAV9-TIE-shRNA (cGAS) into the SFO of mice nor the empty virus vector promoted neuroinflammation in the SFO. E. Neither the SFO-specific knockout of endothelial cGAS by injection of AAV9-TIE-shRNA (cGAS) into the SFO of mice nor the empty virus vector promoted sympathetic activation. *n* = 8, *P* < 0.05, ANOVA LSD test. **Figure S6.** Plasmic NE level was measured in mice. *n* = 8, *P* < 0.05, ANOVA LSD test. **Figure S7.** Heart rate and mean artery pressure were measured in mice. *n* = 8. ANOVA LSD test.

## Data Availability

All relevant data are within the manuscript and Additional file.

## Abbreviations

C-Mito: Circulating mitochondria
HF: Heart failure
SFO: Subfornical organ
ECs: Endothelial cells
cGAS: Cyclic GMP–AMP synthase
ISO: Isoprenaline
DHODH: Dihydroorotate dehydrogenase
KD: Knockdown
dsDNA: Double-strand deoxyribonucleic acid
RASi: Renin-angiotensin system inhibitors
SGLT2i: Sodium–glucose co-transporter 2 inhibitors
PVN: Paraventricular nucleus
RVLM: Rostral ventrolateral medulla
ROS: Reactive oxygen species
STING: Stimulator of interferon genes protein
NLRP3: NLR family pyrin domain containing 3
mtDNA: Mitochondrial deoxyribonucleic acid
iCAM: Intercellular adhesion molecule 1
ELISA: Enzyme-linked immunosorbent assay
HRV: Heart rate variability
RSNA: Renal sympathetic nerve activity
WGA: Wheat Germ Agglutinin
