# Supplementary material for: Circulating mitochondria promoted endothelial cGAS-derived neuroinflammation in subfornical organ to aggravate sympathetic overdrive in heart failure mice
Source: J Neuroinflammation. 2024 Jan 19;21:27. doi: 10.1186/s12974-024-03013-x (PMC10799549; doi:10.1186/s12974-024-03013-x)
Supplement: Supplementary file 1 — Additional file 1: Figure S1. The expression of mitochondrial respiratory complexes (A), mitochondrial outer membrane protein TOM20, inner membrane protein TIM50, and matrix protein Hsp60 (B) were detected by immunoblotting in order to verify that the isolated microparticles were C-Mito. n = 8. Figure S2. Immunofluorescent staining was conducted to detect the co-localization of C-Mito and neurons (A, B), astroglia (C, D), and microglia (E, F). n = 8. ANOVA LSD test. Figure S3. Immunofluorescent staining was conducted to detect the co-localization of cGAS and neurons (A, B), astroglia (C, D), and microglia (E, F). n = 8. ANOVA LSD test. Figure S4. pre-treated with Terflunomide on C-MitoHF mitigated the cGAS-upregulating effect of C-MitoHF in ECs of the SFO in HF mice in vivo. n = 8, P < 0.05, t test. Figure S5. Verification of endothelial cGAS KD in the SFO of mice by AAV9-TIE-shRNA (cGAS) injection. A. The co-localization of AAV9 virus and ECs in the SFO of mice. Scale bar = 200 μm. B, C. Immunofluorescent staining showed successful endothelial cGAS KD in the SFO of mice. Scale bar = 100 μm. D. Neither the SFO-specific knockout of endothelial cGAS by injection of AAV9-TIE-shRNA (cGAS) into the SFO of mice nor the empty virus vector promoted neuroinflammation in the SFO. E. Neither the SFO-specific knockout of endothelial cGAS by injection of AAV9-TIE-shRNA (cGAS) into the SFO of mice nor the empty virus vector promoted sympathetic activation. n = 8, P < 0.05, ANOVA LSD test. Figure S6. Plasmic NE level was measured in mice. n = 8, P < 0.05, ANOVA LSD test. Figure S7. Heart rate and mean artery pressure were measured in mice. n = 8. ANOVA LSD test. [file 12974_2024_3013_MOESM1_ESM.docx]

**Additional file 1**

**Figure S1**


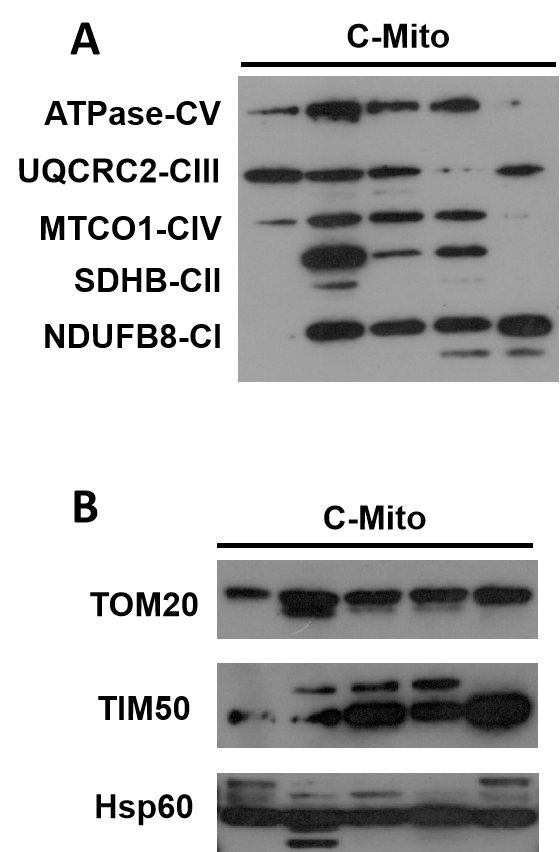


Figure S1. The expression of mitochondrial respiratory complexes (A), mitochondrial outer membrane protein TOM20, inner membrane protein TIM50, and matrix protein Hsp60 (B) were detected by immunoblotting in order to verify that the isolated microparticles were C-Mito. n = 8.

**Figure S2**


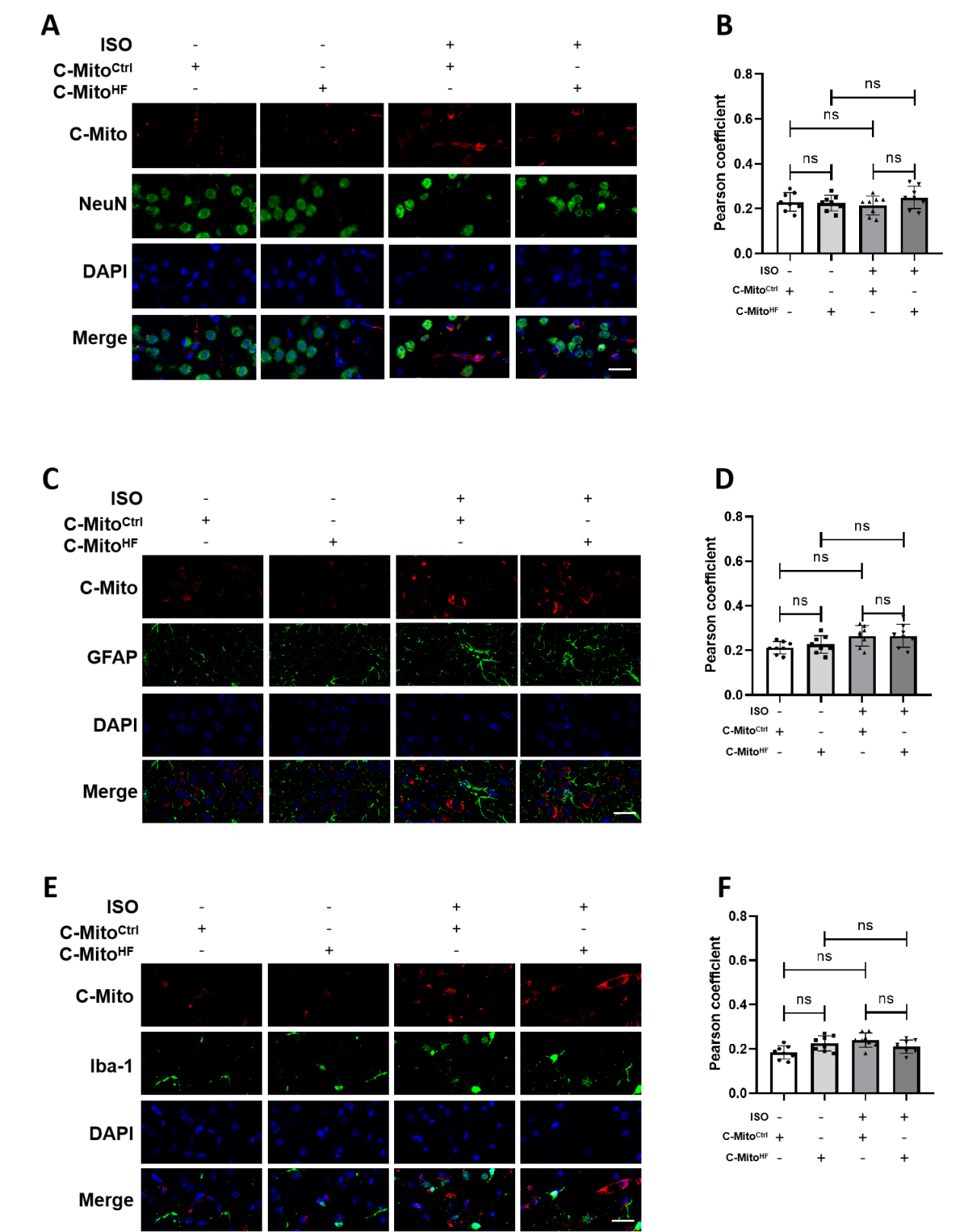


Figure S2. Immunofluorescent staining was conducted to detect the co-localization of C-Mito and neurons (A-B), astroglia (C-D), and microglia (E-F). n = 8. ANOVA LSD test.

**Figure S3**


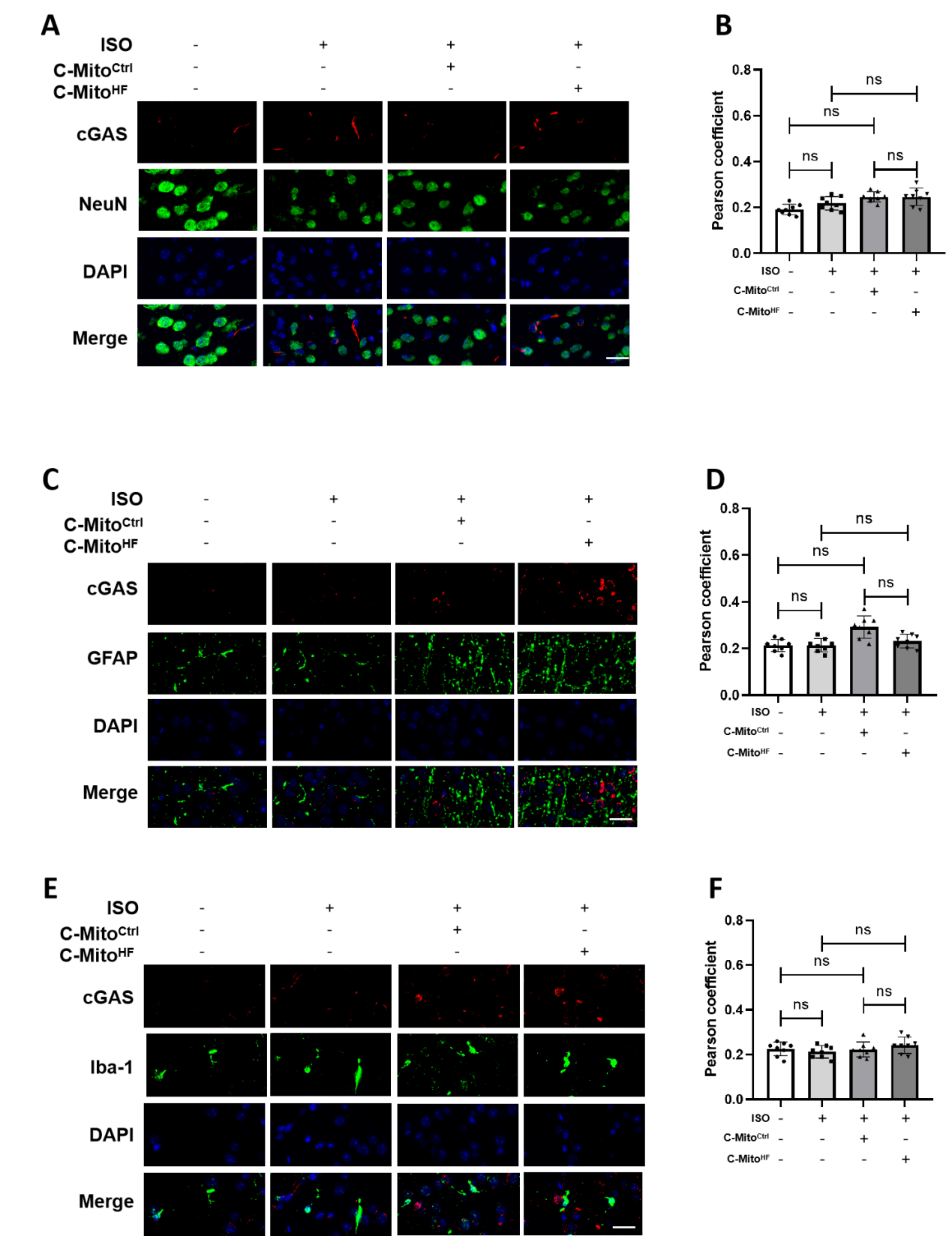


Figure S3. Immunofluorescent staining was conducted to detect the co-localization of cGAS and neurons (A-B), astroglia (C-D), and microglia (E-F). n = 8. ANOVA LSD test.

**Figure S4**


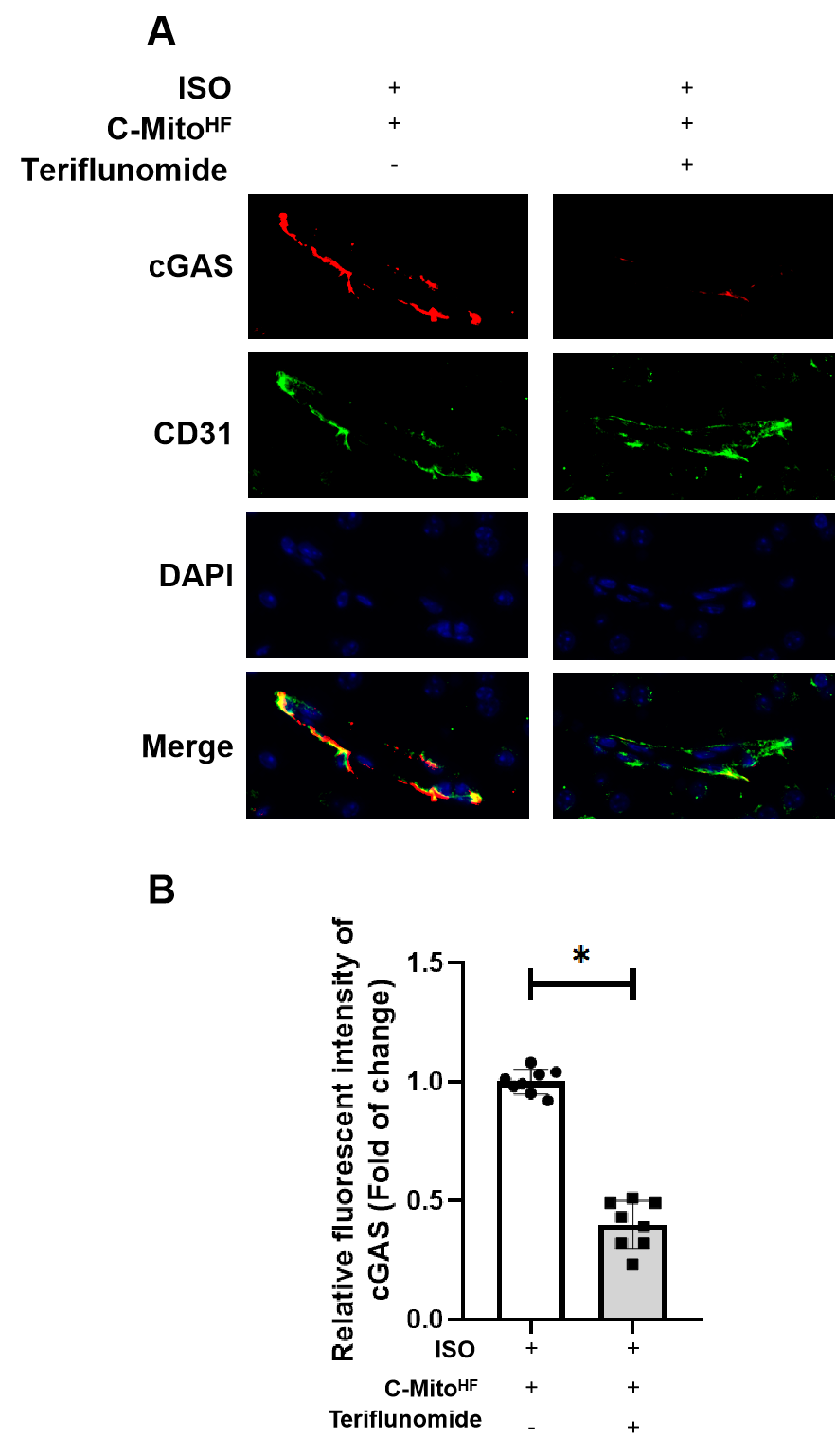


Figure S4. pre-treated with Terflunomide on C-Mito^HF^ mitigated the cGAS-upregulating effect of C-Mito^HF^ in ECs of the SFO in HF mice in vivo. n = 8, *P* < 0.05, *t* test.

**Figure S5**


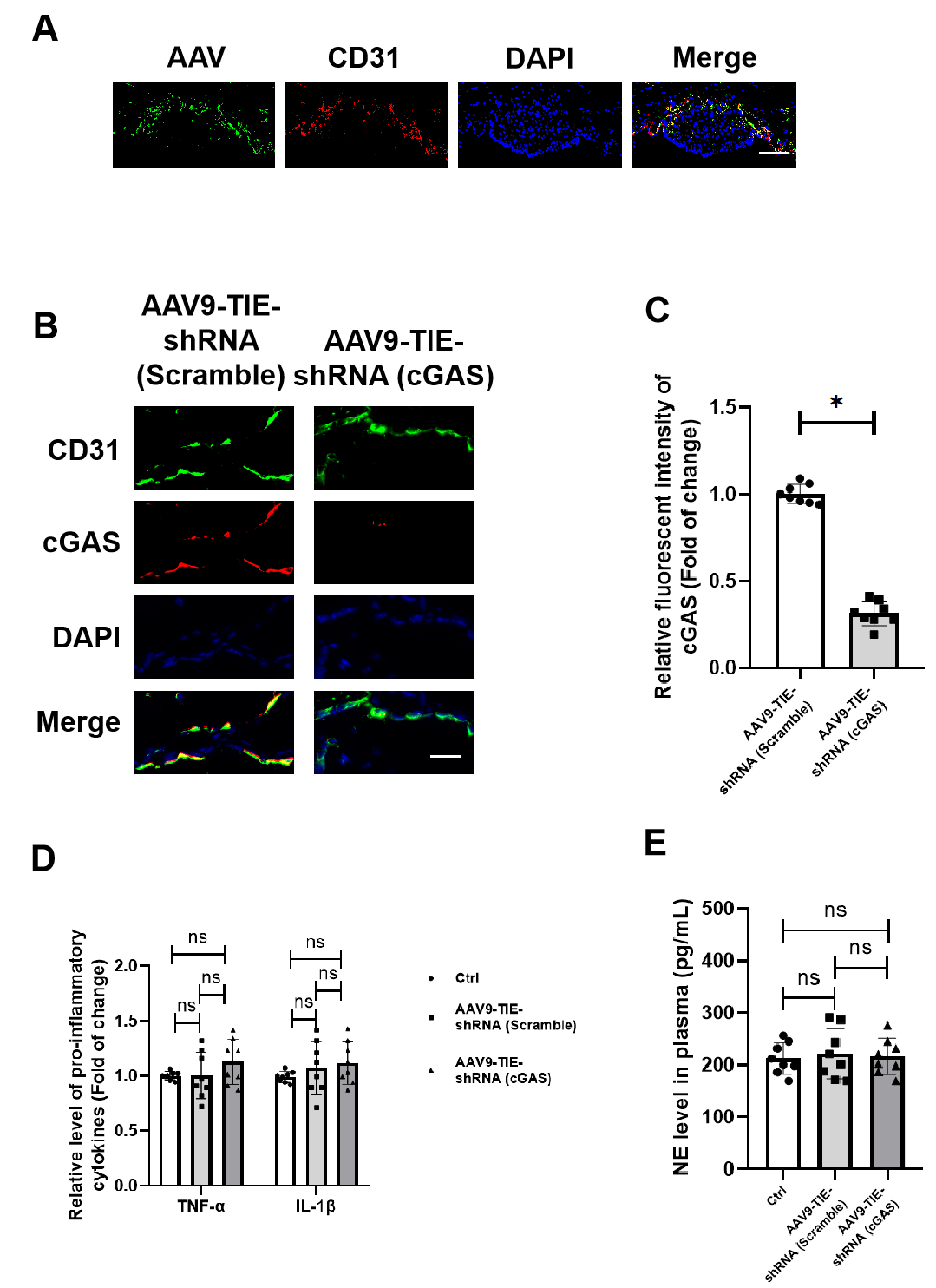


Figure S5. Verification of endothelial cGAS KD in the SFO of mice by AAV9-TIE-shRNA (cGAS) injection. A. The co-localization of AAV9 virus and ECs in the SFO of mice. Scale bar = 200 μm. B-C. Immunofluorescent staining showed successful endothelial cGAS KD in the SFO of mice. Scale bar = 100 μm. D. Neither the SFO-specific knockout of endothelial cGAS by injection of AAV9-TIE-shRNA (cGAS) into the SFO of mice nor the empty virus vector promoted neuroinflammation in the SFO . E. Neither the SFO-specific knockout of endothelial cGAS by injection of AAV9-TIE-shRNA (cGAS) into the SFO of mice nor the empty virus vector promoted sympathetic activation. n = 8, *P* < 0.05, ANOVA LSD test.

**Figure S6**

**
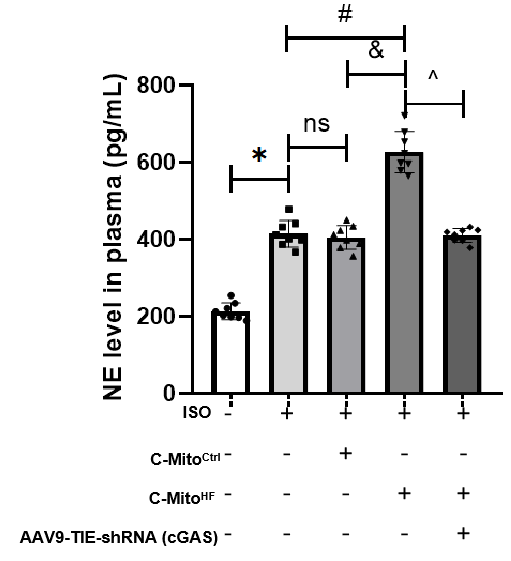
**

Figure S6. Plasmic NE level was measured in mice. n = 8, *P* < 0.05, ANOVA LSD test.

Figure S7


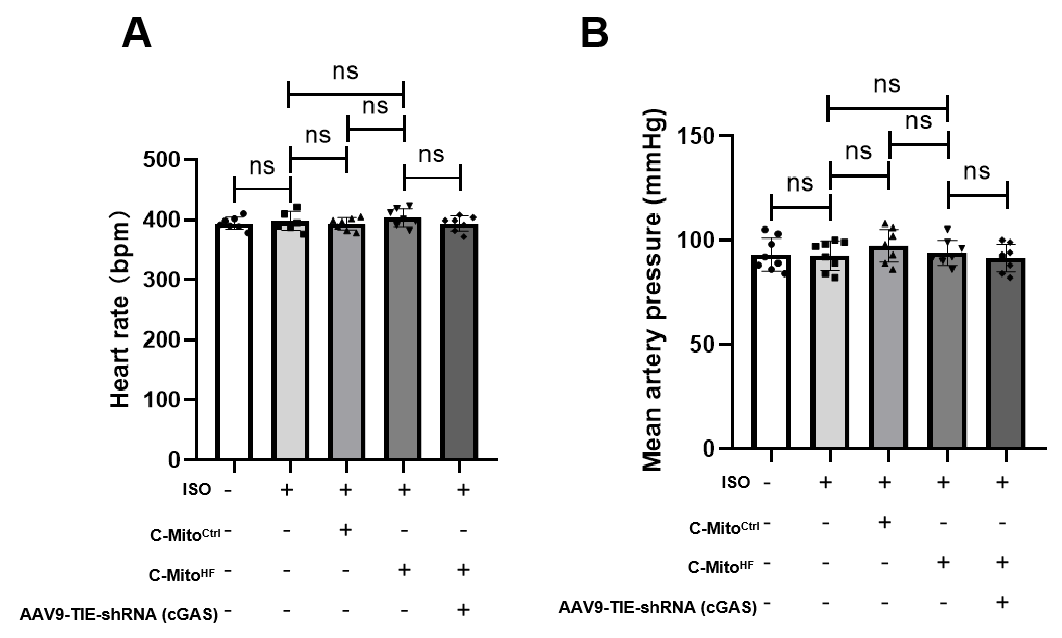


Figure S7. Heart rate and mean artery pressure were measured in mice. n = 8. ANOVA LSD test.
